# Silibinin-derived microbiota enrich (R)-2,3-dihydroxy-isovalerate and ameliorate colitis via the GAT-3/RARβ/RORγt axis

**DOI:** 10.1093/ismejo/wraf175

**Published:** 2025-08-13

**Authors:** Baofei Yan, Xian Zheng, Danya Lu, Ting Li, Xi Chen, Zhitao Shao, Tingming Fu

**Affiliations:** State Key Laboratory on Technologies for Chinese Medicine Pharmaceutical Process Control and Intelligent Manufacture, Nanjing University of Chinese Medicine, Nanjing 210023, P. R. China; Department of Pharmacy, Affiliated Kunshan Hospital of Jiangsu University, Kunshan 215399, P. R. China; State Key Laboratory on Technologies for Chinese Medicine Pharmaceutical Process Control and Intelligent Manufacture, Nanjing University of Chinese Medicine, Nanjing 210023, P. R. China; State Key Laboratory on Technologies for Chinese Medicine Pharmaceutical Process Control and Intelligent Manufacture, Nanjing University of Chinese Medicine, Nanjing 210023, P. R. China; Jiangsu Engineering Research Center for Precision Prevention and Treatment of Digestive and Reproductive System Tumors, Huaian 223003, P. R. China; State Key Laboratory on Technologies for Chinese Medicine Pharmaceutical Process Control and Intelligent Manufacture, Nanjing University of Chinese Medicine, Nanjing 210023, P. R. China; State Key Laboratory on Technologies for Chinese Medicine Pharmaceutical Process Control and Intelligent Manufacture, Nanjing University of Chinese Medicine, Nanjing 210023, P. R. China

**Keywords:** ulcerative colitis, silibinin, gut microbiota, (R)-2,3-dihydroxy-isovalerate, GAT-3/RARβ/RORγt axis

## Abstract

Microbiota-associated factors are increasingly recognized as significant contributors to the progression of ulcerative colitis, and microbial modulation has emerged as an effective therapy for this condition. The herbal compound silibinin has demonstrated properties that modulate gut microbiota. Herein, we investigated the response of gut microbiota to silibinin in ameliorating colitis, using a mouse model of colitis coupled with antibiotic exposure. Results indicated that antibiotic pretreatment negated the benefits of silibinin in mice with colitis. Furthermore, fecal microbiota transplantation involving silibinin-modulated gut microbiota further substantiated the gut microbiota-dependent effects of silibinin. Within the metabolic profiles of silibinin-regulated microbiota, we identified that *Alistipes*-associated (R)-2,3-dihydroxy-isovalerate exhibited the most pronounced anti-inflammatory effects *in vitro* and demonstrated protective effects against colitis. Moreover, (R)-2,3-dihydroxy-isovalerate reinstated the protective effects of silibinin in mice with colitis under antibiotic exposure. These effects were primarily mediated via the targeting of the colonic GABA transporter 3 (GAT-3) by (R)-2,3-dihydroxy-isovalerate. We further revealed that the retinoic acid receptor β and the retinoid-related orphan nuclear receptor γt may mediate the impact of silibinin-derived microbiota and (R)-2,3-dihydroxy-isovalerate on colitis. Additionally, the knockdown of colonic GAT-3 diminished the impact of silibinin on the GAT-3/retinoic acid receptor β/retinoid-related orphan nuclear receptor γt axis and colitis. Our findings highlight that (R)-2,3-dihydroxy-isovalerate, enriched from microbiota derived from silibinin, can target the GAT-3/retinoic acid receptor β/retinoid-related orphan nuclear receptor γt axis, which is essential for anti-colitis properties of silibinin-regulated microbiota.

## Graphical Abstract

**Graphical Abstract:**
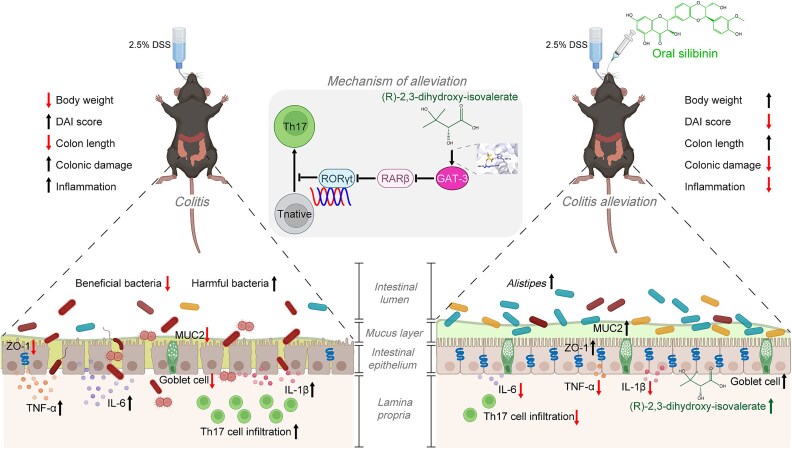

## Introduction

Ulcerative colitis (UC) is a chronic inflammatory bowel disease (IBD) distinguished by symptoms such as abdominal pain, diarrhea, and rectal bleeding [1]. Together with Crohn’s disease, it is collectively known as IBD [1]. The typical progression of UC begins in the distal rectum and progressively extends throughout the colon, with the potential to affect the entire organ [1]. Prolonged inflammatory infiltration of the colon is recognized as one of the significant risk factors for the development of colorectal cancer [2]. At present, the incidence of UC is exhibiting a steady increase on a global scale each year [1]. There are currently no effective treatments available for UC. Common treatments used in clinical practice, such as corticosteroids and immunosuppressants, may lead to drug resistance and adverse effects with prolonged use. Additionally, discontinuing these medications can result in disease relapse and exacerbation [3]. As UC progresses uncontrollably and becomes unresponsive to medical management, invasive surgical intervention is frequently employed, despite its association with considerable physical and psychological distress for patients [4]. Therefore, there is an urgent need to identify novel, effective, and highly adaptable therapeutic strategies for UC.

The pathogenesis of UC remains poorly understood. However, it is widely recognized that key contributing factors encompass genetic susceptibility, dysfunction of the epithelial barrier, dysregulation of immune responses, microbial dysbiosis, and environmental influences [1]. Among these, the gut microbiota have received increasing attention. The gut microbiota are often regarded as the largest “metabolic organ” of the body, with the interactions between the host and gut microbiota playing a critical role in the occurrence and development of UC [5]. Clinical studies have demonstrated that the diversity of gut microbiota in UC patients is significantly altered, exhibiting a pronounced imbalance between beneficial and harmful bacterial populations [6]. The gut microbiota dysbiosis has been shown to positively correlate with the enhanced differentiation of Th17 cells and the increased secretion of pro-inflammatory factors, both of which contribute to the induction of intestinal mucosal inflammatory responses [7]. In addition, these dysbiotic alterations may be associated with an increase in bacterial toxins and a reduction in beneficial microbiota-derived metabolites [3]. Reportedly, elevated levels of lipopolysaccharide (LPS) in the intestines of UC patients and experimental animal models can directly activate inflammatory signaling pathways, thereby inducing intestinal inflammation [8]. Supplementation with butyrate, a short-chain fatty acid (SCFA) produced by gut microbiota, has been found to facilitate the restoration of intestinal mucosal barrier function [9]. Numerous studies have supported that supplementation with probiotics and prebiotics, as well as fecal microbiota transplantation (FMT) from healthy individuals, can effectively ameliorate UC [10]. Together, current studies suggest that gut microbiota represent a viable therapeutic target for UC, and microbiota-derived metabolites play an important mediating role.

Silibinin, the primary active polyphenolic flavonoid of *Silybum marianum*, is frequently employed as an adjunctive therapy for liver diseases in clinical settings and as a dietary nutritional supplement [11]. In addition, silibinin functions as a modulatory agent of the gut microbiota, presenting potential for the restoration of dysregulated microbial communities and thereby treat diseases. In a clinical trial, silymarin, with silibinin as its main component, may improve liver stiffness in patients with metabolic dysfunction-associated steatotic liver disease (MASLD) through modulation of gut microbiota [12]. Another study reported that the lipid-lowering effect of silymarin was attributed to silymarin-induced bacterial B12 production, which was validated by mouse microbiome depletion and FMT experiments [13]. In our previous studies, we identified that the therapeutic benefits of silibinin in mitigating high-fat diet-induced MASLD mice were associated with the remodeling of gut microbiota diversity [11, 14]. Moreover, silibinin has been shown to modulate metabolites of the gut microbiota such as SCFAs and bile acids, which indirectly enhance its pharmacological effects [15]. In this study, we report a significant modulation of gut microbiota in mice with dextran sulfate sodium (DSS)-induced UC following the administration of silibinin. The subsequent enrichment of (R)-2,3-dihydroxy-isovalerate contributed to the amelioration of UC through the GABA transporter 3 (GAT-3)/retinoic acid receptor β (RARβ)/retinoid-related orphan nuclear receptor γt (RORγt) axis.

## Materials and methods

### Drugs and reagents

Silibinin, with a purity of no less than 98%, was procured from Aladdin (Shanghai, China). DSS (molecular weight: 36–50 kDa) was supplied by MP Biomedicals (CA, USA). Mesalazine (Mes) was obtained from Houston, USA. (R)-2,3-dihydroxy-isovalerate with a purity ≥98% was sourced from Zhenglesheng (Suzhou, China). Antibiotics including ampicillin, streptomycin, and colistin were acquired from Wako Chemicals (VA, USA).

### Bacterial culture

*Alistipes indistinctus* and *Alistipes finegoldii* were obtained from Biobw (Beijing, China) and cultured in BHI broth (Hopebio, Qingdao, China) at 37°C under anaerobic conditions. For oral gavage, *A. indistinctus* and *A. finegoldii* were harvested during the logarithmic growth phase by centrifugation. The bacterial pellets were then washed and resuspended in PBS to achieve a final concentration of 1 × 10^9^ CFU/ml.

### Animals

Male C57BL/6 mice aged 6 weeks (body weight: 20–24 g) were sourced from GemPharmatech (Nanjing, China) for this study. The animals were housed at the experimental animal center of Nanjing University of Chinese Medicine under well-controlled environmental conditions, including a temperature range of 24°C–26°C, relative humidity maintained at 40%–50%, and a 12-h light–dark cycle. Food and water were provided *ad libitum* throughout the experimental period. All procedures involving animal care adhered to the guidelines established by the European Community, and the experimental protocols were approved by the animal ethics committee of Nanjing University of Chinese Medicine (approval no. 202310A021).

### Animal grouping and treatment

Following a 7-day acclimation period, mice were evenly and randomly distributed into distinct experimental groups (*n* = 10/group). Our previous findings indicate that mice given free access to 2.5% (w/v) DSS for 10 days developed significant clinical symptoms of UC, whereas maintaining a high survival rate [3, 16]. Therefore, UC was induced by administering 2.5% (w/v) DSS in the drinking water for 10 consecutive days (DSS group). Simultaneously, mice received daily oral gavage of either Mes (310 mg/kg), silibinin at doses of 20, 50, or 100 mg/kg, (R)-2,3-dihydroxy-isovalerate (50 mg/kg), or 0.2 ml of bacterial suspension from day 1 to day 10 (intervention groups). Drawing on previous research and our preliminary findings, we selected oral doses of 20, 50, and 100 mg/kg of silibinin [17]. Mes dose was adjusted from its clinically approved regimen. Additionally, our preliminary experiments demonstrated that (R)-2,3-dihydroxy-isovalerate was nontoxic to mice at doses up to 150 mg/kg. Consequently, we chose a dose of 50 mg/kg for (R)-2,3-dihydroxy-isovalerate, consistent with the silibinin dosage used in this study. Mice, which underwent no specific treatment, were instead gavaged daily with normal saline (control group). Fresh fecal pellets were collected from donor mice with DSS-induced UC that had been treated with 50-mg/kg silibinin. These fecal samples were pooled, diluted in sterile PBS (0.1 g of feces per 1 ml of PBS), homogenized, and filtered through a 100-μm strainer for subsequent applications.

An additional subset of mice underwent treatment with a broad-spectrum antibiotic (ABX) cocktail, consisting of 1-mg/ml ampicillin, 5-mg/ml streptomycin, and 1-mg/ml colistin, administered via drinking water for a duration of 2 weeks. Following the pretreatment phase, UC was induced using a 2.5% DSS solution provided for 10 days. During the same period, mice were orally gavaged with one of the following: normal saline, 50-mg/kg silibinin, a combination of 50-mg/kg silibinin and 50-mg/kg (R)-2,3-dihydroxy-isovalerate, or 0.2 ml of filtered donor fecal homogenate, or 0.2 ml of bacterial suspension (*n* = 10/group).

Following a 10-day induction with 2.5% DSS, a third subset of mice was administered via oral gavage with one of the following: normal saline, 50-mg/kg silibinin, 50-mg/kg (R)-2,3-dihydroxy-isovalerate, 50-mg/kg silibinin in conjunction with AAV-shGAT-3 pretreatment (Yinrui, Nanjing, China), or 50-mg/kg (R)-2,3-dihydroxy-isovalerate in combination with AAV-shGAT-3 pretreatment (*n* = 10/group). For AAV-shGAT-3 pretreatment, mice received a suspension of AAV-shGAT-3 virus (titer >10^13^) via enema for seven consecutive days. The comprehensive schematic representation of the experimental procedures and treatment regimens is depicted in the corresponding figures.

### Behavioral observation and sample collection

Throughout the experiment, daily assessments were conducted to monitor body weight, occurrences of diarrhea, and the presence of blood in stools, with the disease activity index (DAI) determined using the evaluation criteria outlined in Supplementary Table S1. Following the final treatment, all mice were subjected to a 12-h fasting period, during which water was provided *ad libitum*. Blood samples were subsequently obtained from the retro-orbital venous plexus, incubated at room temperature for 2 h, and centrifuged at 3500 rpm for 10 min to isolate the serum. At last, the mice were euthanized via carbon dioxide overdose. An abdominal incision was made to excise the colon and its contents. The colonic contents, along with a segment of colonic tissue, were rapidly frozen in liquid nitrogen, whereas an additional portion of colonic tissue was preserved in 4% paraformaldehyde for further analyses.

### Histopathological analysis

Fixed colonic tissues were paraffin-embedded, sectioned at a thickness of 5 μm, and stained using hematoxylin and eosin (H&E) and periodic acid-Schiff (PAS) kits (ServiceBio, Wuhan, China) adhering to established protocols. Subsequently, the colonic pathology was characterized in accordance with previously established methodologies, and the quantification of goblet cells within the crypts was conducted [18].

### Enzyme-linked immunosorbent assay

Serum concentrations of IL-6, IL-1β, and tumor necrosis factor α (TNF-α) were determined using enzyme-linked immunosorbent assay (ELISA) kits supplied by ServiceBio, following standard protocols. For colonic tissue analysis, samples were homogenized with an AllSheng Bioprep-24 homogenizer (Hangzhou, China) and subjected to centrifugation at 12000 rpm for 15 min to isolate the supernatants. The levels of IL-6, IL-1β, and TNF-α in the resulting supernatants were quantified using corresponding ELISA kits in accordance with established protocols. Furthermore, treated RAW264.7, NCM460, and Caco-2 cells were harvested, lysed, and centrifuged at 12000 rpm for 15 min to obtain supernatants, which were subsequently analyzed for IL-1β concentrations using commercially available kits as instructed by the manufacturer.

### Immunohistochemistry

Colon tissues were processed by fixation, dehydration, embedding, and slicing into 4-μm sections. Endogenous peroxidase activity was blocked, and antigen retrieval was performed. The tissue sections were incubated overnight at 4°C with primary antibodies diluted to 1:200, which specifically targeted mucin 2 (MUC2, ServiceBio), zonula occludens-1 (ZO-1, ServiceBio), cluster of differentiation 86 (CD86, ServiceBio), GAT-3 (ServiceBio), RARβ (Affinity, Changzhou, China), and RORγt (Affinity). Following primary incubation, sections were exposed to an appropriate secondary antibody (diluted 1:400) for 1 h at room temperature. Color development for immunohistochemical analysis was conducted utilizing the DAB Horseradish Peroxidase Color Development Kit (Beyotime, Shanghai, China). The resulting images were captured using Leica DM2500/DM3000 optical microscopes (Wetzlar, Germany) and subsequently analyzed with Image-Pro Plus 6.0 software (Media Cybernetics, MD, USA).

### *Ex vivo* culture of fecal microbiota

The *ex vivo* experiments were conducted following previously established methods [15]. In brief, colonic contents from DSS-induced UC mice were suspended in sterilized anaerobic PBS to create 20% (w/v) slurries within an anaerobic incubator (Immanuel Instrument, Nanjing, China). The slurries underwent homogenization and subsequent centrifugation to isolate the supernatant, which contained the fecal microbiota. Prior to anaerobic incubation at 37°C for 48 h, 1 ml of the supernatant was mixed with 9 ml of GAM medium (Hopebio) enriched with vitamin K1 and heme. The mixture was then further supplemented with silibinin to achieve final concentrations of 0, 100, and 200 μM.

### Gut microbiota sequencing

Total DNA was extracted from colonic contents and fecal microbiota fermentation broth utilizing the QIAamp DNA Stool Mini Kit (Qiagen, Hilden, Germany), in strict accordance with the manufacturer’s protocols. To amplify the V3-V4 region of the 16S rRNA gene, universal primers 338F (5′-ACTCCTACGGGAGGCAGCA-3′) and 806R (5′-GGACTACHVGGGTWTCTAAT-3′) were employed. The resulting amplicons underwent purification via the AxyPrep DNA Gel Extraction Kit (CA, USA) and were quantified using the Promega QuantiFluor-ST fluorometer (WI, USA). Sequencing libraries were subsequently constructed with the Thermo Fragment Library Kit (MA, USA) and sequenced on the MiSeq PE300 platform (Illumina, TX, USA).

The sequencing analysis process is as follows. Raw FASTQ files were quality-filtered using FASTP (v0.20.0) and merged with FLASH (v1.2.7) under the following criteria. (i) Reads were truncated at any site where the average quality score dropped below 20 within a 50-bp sliding window. (ii) Paired-end reads with overlaps longer than 10 bp were merged, allowing a maximum of two mismatches. Sequences from each sample were demultiplexed based on exact barcode matches and primer sequences allowing up to two mismatches; reads containing ambiguous bases were removed. Noise reduction was performed using the DADA2 plugin within the QIIME2 pipeline to obtain amplicon sequence variants (ASVs) from the quality-controlled, merged reads. All samples were rarefied to 20 000 sequences. Taxonomic classification of ASVs was performed using the Naive Bayes classifier in QIIME2, referencing the SILVA 16S rRNA gene database (v138).

### Metabolomic analysis of *in vivo* and *ex vivo* fecal microbiota

Characterization of metabolites within colonic contents and fecal microbiota fermentation broth was performed based on previous reports with slight modifications [3]. For this analysis, colonic tissue and fecal microbiota fermentation broth samples were first homogenized and subsequently subjected to centrifugation to yield the supernatant, which was mixed with methanol and dried under nitrogen gas. The resulting dried extracts were reconstituted, filtered, and subjected to analysis using the Vanquish-Orbitrap Exploris 120 liquid chromatography-mass spectrometry (LC–MS, Thermo) system. Both chromatographic and mass spectrometric parameters were meticulously optimized for positive and negative ion modes, thereby enabling high-resolution detection during primary full scans and targeted MS/MS analyses. Metrological analysis of the data was performed utilizing the BioDeep online platform (PANOMIX, Suzhou, China), whereas the Spearman correlation analysis examining the relationships between bacteria and metabolites was carried out on the Majorbio online platform.

### Cell culture and treatment

RAW264.7, NCM460, and Caco-2 cells, sourced from the Chinese Academy of Cell Resource Center (Shanghai, China), were cultured in either DMEM or 1640 Medium (KeyGEN, Nanjing, China) supplemented with 10% fetal bovine serum (FBS, Bio-Channel, Nanjing, China) at 37°C in a humidified atmosphere of 5% CO₂. To ascertain the safe concentration of the selected metabolites for RAW264.7, NCM460, and Caco-2 cells, a CCK8 kit (Beyotime) was performed in accordance with the manufacturer’s guidelines. Preliminary experiments established that a concentration of 1 μg/ml LPS (MCE, Shanghai, China) and 20-ng/ml TNF-α (PeproTech, Suzhou, China) effectively induced inflammation *in vitro*. For treatment interventions, cells placed in serum-free medium were exposed to a concentration of 50 μM of the selected metabolites for 24 h in the presence of 1-μg/ml LPS or 20 ng/ml of TNF-α.

### Detection of colonic (R)-2,3-dihydroxy-isovalerate

Colonic tissues of mice administered with *A. indistinctus* and *A. finegoldii* were prepared following established metabolomics pretreatment protocols. Subsequently, the relative abundance of (R)-2,3-dihydroxy-isovalerate was quantified utilizing the Triple Quad 5500 LC–MS system (AB SCIEX, MA, USA). For further details, please refer to the Supporting material.

### Colon transcriptome sequencing

Total RNA was extracted from colon tissues, followed by an assessment of its quality and concentration utilizing a NanoPhotometer spectrophotometer (IMPLEN, CA, USA) and an Agilent Bioanalyzer 2100 system, respectively. Subsequently, an RNA sequencing library was constructed employing an RNA Library Prep kit, and sequencing was performed on the NovaSeq 6000 system (Illumina). The acquired raw data underwent a series of processing, filtering, and sequencing analysis on the Majorbio online platform.

### Prediction of targets for (R)-2,3-dihydroxy-isovalerate

To obtain the predicted targets, structural information of (R)-2,3-dihydroxy-isovalerate (PubChem CID: 440279) was retrieved from PubChem (https://pubchem.ncbi.nlm.nih.gov/) and subsequently imported into SwissTargetPrediction (http://www.swisstargetprediction.ch/).

### Molecular docking

The crystal structures of GAT-3 (AlphaFoldDB Code: P48066), RARβ (PDB Code: 4DM8), and RORγt (PDB Code: 4NB6) were sourced from the AlphaFold Data Bank (https://alphafold.ebi.ac.uk) and the Protein Data Bank (https://www.rcsb.org/). Prior to analysis, the protein structures underwent preprocessing steps that encompassed hydrogenation, charging, and protonation, facilitated by the Protein Preparation Wizard module in Schrödinger (NY, USA), which was kindly operated by Dr. Li Liang from China Pharmaceutical University (Nanjing, China). The compound (R)-2,3-dihydroxy-isovalerate (PubChem CID: 440279) underwent protonation and subsequent energy minimization through the application of the LigPrep module from Schrödinger. Binding sites for the preprocessed protein structures were predicted utilizing the Sitemap module within the Schrödinger suite, leading to the generation of docking grid files for the most promising predicted sites. Finally, interaction visualization was performed employing PyMOL software (Schrödinger).

### Molecular dynamics simulation

The tleap module within AMBER v22 software (CA, USA) was employed to incorporate the missing hydrogen atoms into the complex and concurrently generating partial charges and parameters for the ligand. To achieve system neutrality, a tailored quantity of Na^+^ or Cl^−^ ions was introduced. The process commenced with energy minimization, after which the system was gradually heated from 0 to 300 K over a span of 300 ps. To optimize the balance between docking speed and accuracy, a molecular dynamics simulation of 100 ns was conducted at a constant temperature of 300 K, utilizing a time step of 2.0 fs.

### Western blotting

Colonic samples were subjected to lysis in prechilled RIPA buffer, enriched with a protease inhibitor cocktail. The total protein concentration was then quantified using the BCA assay. Primary antibodies—specifically directed against GAT-3, RARβ, RORγt, and glyceraldehyde-3-phosphate dehydrogenase (GAPDH, ServiceBio)—were diluted to a concentration of 1:1000 and incubated with the protein bands overnight at 4°C. The protein bands were labeled with secondary antibodies for a period of 1 h in the dark. Band visualization and semiquantitative analysis were conducted utilizing the Azure Biosystems C600 (CA, USA) and ImageJ software (NIH, Bethesda, MD, USA), respectively.

### Cellular thermal shift assay

Cells were subjected to lysis using precooled PBS. The resulting lysates were subsequently divided into two aliquots, which were treated for 1 h at room temperature with either DMSO or (R)-2,3-dihydroxy-isovalerate. Following the treatment, each lysate was heated separately at specified temperatures ranging from 37°C to 60°C for 5 min and subsequently allowed to cool to ambient temperature. After cooling, the lysates were centrifuged, and the supernatants obtained were analyzed via western blotting.

### Drug affinity responsive target stability

The cell lysates were incubated with (R)-2,3-dihydroxy-isovalerate for a duration of 30 min at room temperature. Following this, pronase E (Merck, Shanghai, China) was introduced at a dilution ranging from 1:1000 to 1:16000, and the samples were subjected to incubation at 40°C for an additional 30 min. Upon cooling, the lysates underwent centrifugation, and the resulting supernatants were subsequently analyzed using western blotting.

### Flow cytometry

As previously detailed [19], single-cell suspensions derived from the colonic lamina propria of mice were subjected to staining with a fluorochrome-conjugated monoclonal anti-IL-17 antibody (CST, MA, USA). Concurrently, the samples were treated with a Dead Cell Stain Kit (Thermo) to identify nonviable cells. Subsequently, the stained samples were processed using a MoFlo XDP ultrafast flow cytometer (Beckman, IN, USA) and analyzed with FlowJo v10 software (OR, USA).

### Protein–protein interaction

The proteins GAT-3 and RORγt were analyzed for their protein–protein interaction relationships using the GeneMANIA database (http://genemania.org/).

### Co-immunoprecipitation assay

Cells were harvested and lysed using prechilled RIPA buffer supplemented with a protease inhibitor cocktail. The lysates were subjected to high-speed centrifugation at 4°C, and the resulting supernatants were collected. A portion of the supernatant was reserved as the input control, whereas the remainder was incubated with an anti-GAT-3 antibody (Abcam, Cambridge, UK) at 4°C overnight, following the manufacturer’s instructions. Magnetic beads (Merck) were then added to the antibody–protein mixture according to the protocol, followed by gentle mixing and incubation at room temperature for 30 min. The beads were isolated using a magnetic rack, and standard washing and elution steps were subsequently performed. An isotype-matched IgG antibody was processed in parallel as a negative control. Protein concentrations were quantified for each group, and the immunoprecipitated samples were analyzed by western blotting.

### Statistical analysis

Data analysis was performed utilizing SPSS version 21.0 (IBM, Armonk, USA). Statistical differences among groups were analyzed using one-way ANOVA, followed by Bonferroni post hoc testing to minimize Type I errors. A significance threshold of *P* < 0.05 was applied after Bonferroni correction. In instances where two groups were compared, a *t*-test was applied.

## Results

### Silibinin-regulated gut microbiota conferred protection against DSS-induced UC

**Figure 1:**
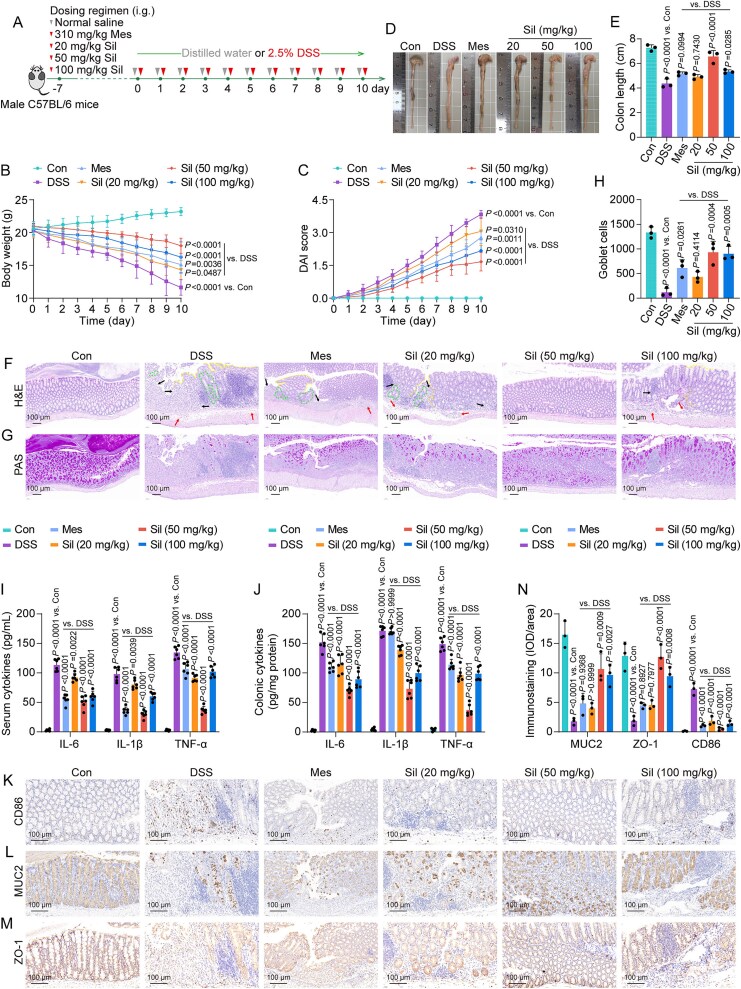
Silibinin alleviated DSS-induced colitis by modulating inflammation, restoring intestinal mucosal architecture, and improving immunological functions. (A) Experimental schedule. (B, C) Dynamic curves of body weight changes and DAI scores. (D, E) phenotypic observations of colons and measurements of colon length. (F, G) H&E (arrows indicate inflammatory cell infiltration or mucosal swelling; dashed lines denote crypt distortion or irregularities on the crypt surface) and PAS staining micrographs. (H) Quantitative analysis of goblet cells in PAS staining. (I, J) Levels of IL-6, IL-1β, and TNF-α in serum and colon lysates. (K-N) Representative immunohistochemical images and corresponding quantitative analyses of CD86, MUC2, and ZO-1 staining intensity in colonic sections. Data are presented as the mean ± SD (*n* = 3–6). *P*-values for each comparison were indicated.

We employed a DSS-induced model of UC to conduct an examination of the potential role of silibinin in alleviating UC symptoms (Fig. 1A). During the experimental period, body weights and DAI scores were systematically recorded and assessed. DSS treatment resulted in a marked reduction in body weights accompanied by a significant elevation in DAI scores in comparison to the control group (Fig. 1B and C). These detrimental effects were effectively ameliorated by silibinin intervention. DSS group exhibited a panoramic view of the colon characterized by pronounced swelling, inflammatory infiltration, and notable shortening when contrasted with the control group (Fig. 1D and E). These pathological features showed significant improvement following silibinin intervention, as indicated by the healthier appearance and increased colon length. H&E staining demonstrated pronounced disruption of the colonic crypt architecture, accompanied by submucosal edema and notable inflammatory infiltration in the DSS group (Fig. 1F). Treatment with silibinin significantly ameliorated these histopathological alterations. PAS staining revealed a substantial decrease in the number of goblet cells present in the DSS group, whereas silibinin treatment led to a remarkable recovery in goblet cell counts (Fig. 1G and H). In the serum and colon of mice subjected to DSS challenge, concentrations of the inflammatory cytokines IL-6, IL-1β, and TNF-α were markedly elevated relative to the control group, suggesting a pronounced inflammatory reaction (Fig. 1I and J). By contrast, treatment with silibinin significantly mitigated the inflammatory responses. There was a marked increase in the infiltration of CD86-positive macrophages, as evidenced by brown staining in the colonic tissues in the DSS treatment group. However, this elevation was significantly diminished upon treatment with silibinin (Fig. 1K and N). Immunostaining for MUC2 and ZO-1 revealed a substantial reduction in intensity (indicated by brown staining) in the DSS group relative to the control group (Fig. 1L–N), highlighting the compromised mucosal integrity. Treatment with silibinin resulted in a marked restoration of these key markers. Taken together, these findings indicated that silibinin exerted extensive ameliorative effects on symptoms associated with DSS-induced UC, evidenced by a reduction in weight loss, diminished DAI scores, decreased colonic inflammation and structural damage, as well as enhanced intestinal immune regulation. Additionally, the therapeutic impact of silibinin did not conform to a dose-dependent relationship; rather, the most pronounced efficacy against UC was recorded at a dosage of 50 mg/kg, whereas a higher dosage of 100-mg/kg yielded diminished results. Furthermore, the therapeutic effect of silibinin at 50 mg/kg exceeded that of Mes. Accordingly, we selected 50 mg/kg of silibinin to conduct our follow-up experiments.

**Figure 2:**
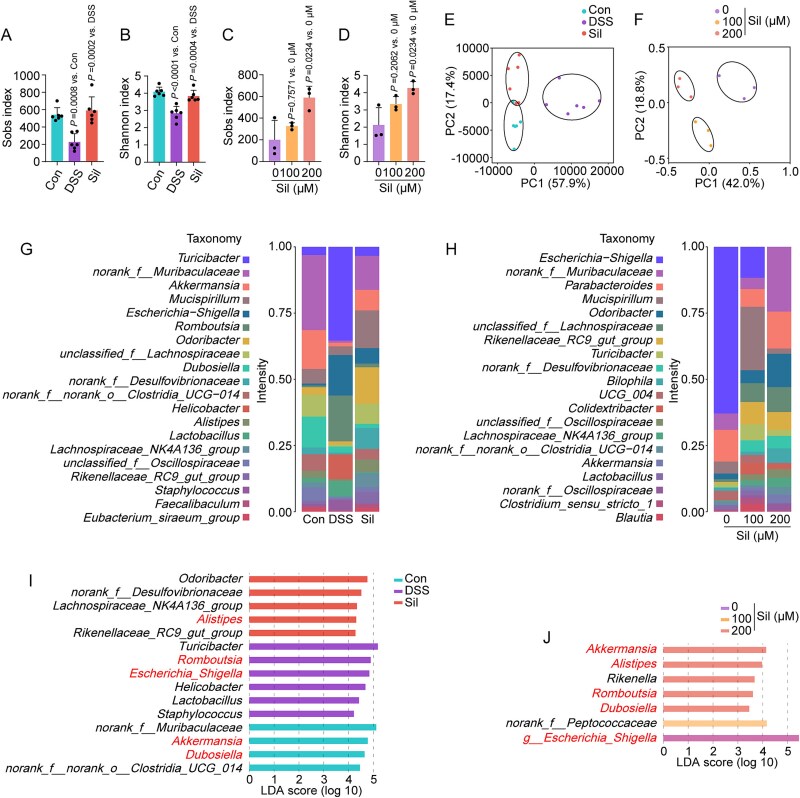
Silibinin corrected the composition of the gut microbiota in DSS-induced mice. (A–F) Sobs and Shannon indices, along with PCA plots of the gut microbiota, were calculated and analyzed for both the experimental mice and the fecal microbiota fermentation broth. (G, H) Relative abundance of bacterial taxa at the genus level in both the experimental mice and the fecal microbiota fermentation broth. (I, J) LefSe analysis identifying significantly different bacterial taxa at the genus level in both the experimental mice and the fecal microbiota fermentation broth. Data are presented as the mean ± SD (*n* = 3–6). *P*-values for each comparison were indicated.

To evaluate whether the protective effects of silibinin against UC were mediated by alterations in the gut microbiota, we initially conducted both *in vivo* and *ex vivo* experiments. Alpha diversity is a vital metric for assessing the richness and evenness of microbial populations, whereas beta diversity reflects the variations in microbial community composition [16]. A significant decrease in alpha diversity, as determined by the sobs and Shannon indices, was observed in the DSS group relative to the control group (Fig. 2A and B). This decline was significantly mitigated following treatment with silibinin. Principal component analysis (PCA) of beta diversity demonstrated a marked segregation between the control group and the DSS group (Fig. 2E), indicating a significant impact of DSS on the composition of gut microbiota communities. The administration of silibinin induced a remodeling of these communities, bringing them closer to that characteristic of the control group. The genus-level species richness (top 20) observed in DSS-challenged mice exhibited a marked divergence from that of the control group, whereas silibinin demonstrated a potent capacity to restore these alterations (Fig. 2G). Consistently, silibinin positively impacted both alpha and beta diversity, alongside genus-level species richness within the DSS-induced gut microbiota in *ex vivo* cultures (Fig. 2C, D, F, and  H). To delve deeper into the inter-group differences at the genus level, we employed linear discriminant analysis effect size (LEfSe) to conduct our investigation. The analysis conducted using LEfSe identified a total of 15 microbial biomarkers present *in vivo*, as well as 7 microbial biomarkers detected *ex vivo* (Fig. 2I and J). Among these taxa, *Alistipes*, *Romboutsia*, *Escherichia-Shigella*, *Akkermansia*, and *Dubosiella* were recognized as shared microbial biomarkers, indicating that these bacterial genera may be the basis for the therapeutic effects of silibinin through the gut microbiota.

**Figure 3:**
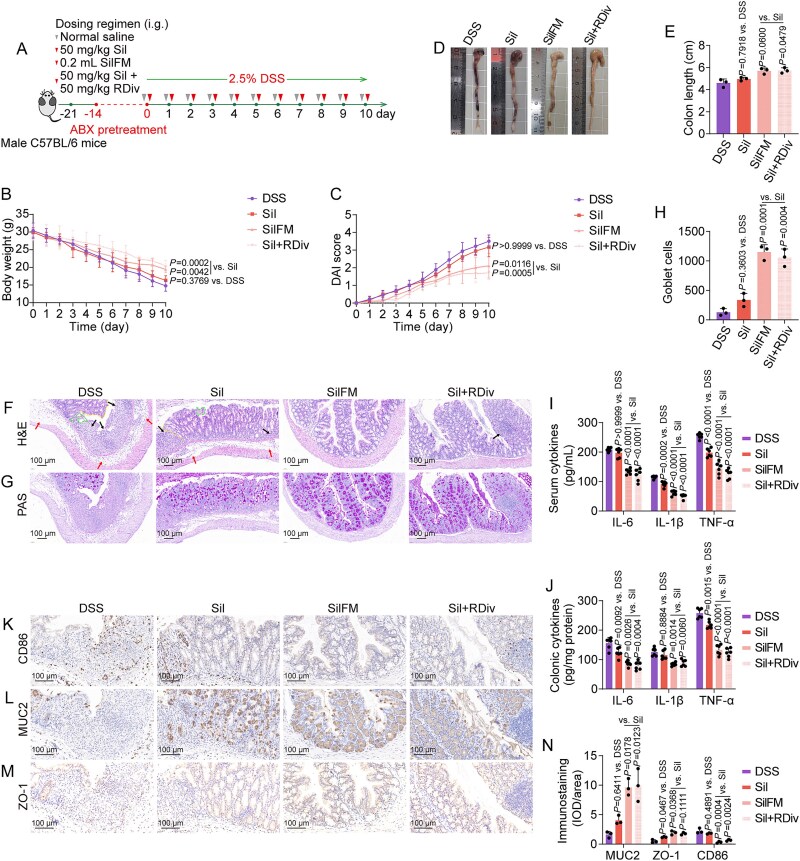
Silibinin-promoted benefits on DSS-induced mice were mediated by the gut microbiota. (A) Schematic representation of the experimental design. (B, C) Dynamic changes in body weight and DAI scores over time. (D, E) Phenotypic observations of colons, including colon length measurements. (F, G) Representative H&E (arrows indicate inflammatory cell infiltration or mucosal swelling; dashed lines denote crypt distortion or irregularities on the crypt surface) and PAS staining micrographs. (H) Quantitative analysis of goblet cells in PAS staining. (I, J) Measured levels of IL-6, IL-1β, and TNF-α in serum and colonic tissue. (K–N) Representative immunohistochemical images and quantification of CD86, MUC2, and ZO-1 staining intensity in the colonic sections. Data are presented as the mean ± SD (*n* = 3–6). *P*-values for each comparison were indicated.

We administered a 2-week course of antibiotic pretreatment to thoroughly deplete gut microorganisms before the DSS challenge, thereby investigating the role of silibinin-regulated gut microbiota in mediating the effects on UC. We conducted FMT experiments, transferring filtered fecal material rich in gut microbiota into pseudo-germ-free mice (Fig. 3A). In pseudo-germ-free mice, silibinin exhibited limited efficacy in alleviating DSS-induced symptoms, including weight loss, increased DAI scores, shortened colon length, and intestinal immune dysregulation induced by DSS. Furthermore, its ability to ameliorate DSS-induced colonic inflammation and structural damage was notably constrained (Fig. 3B–N). The pseudo-germ-free mice challenged with DSS and subsequently treated with fecal microbiota from 50 mg/kg of silibinin-treated mice (SilFM group) exhibited significant improvements, including increased body weight, decreased DAI scores, enhanced colonic length, reduced colonic pathological damage and inflammation, and restored intestinal immune function, compared to those treated exclusively with 50 mg/kg of silibinin. Consequently, silibinin has the potential to ameliorate DSS-induced UC in mice in a gut microbiota-dependent manner.

### Gut microbiota-derived (R)-2,3-dihydroxy-isovalerate conveyed the effects on DSS-induced mice

**Figure 4:**
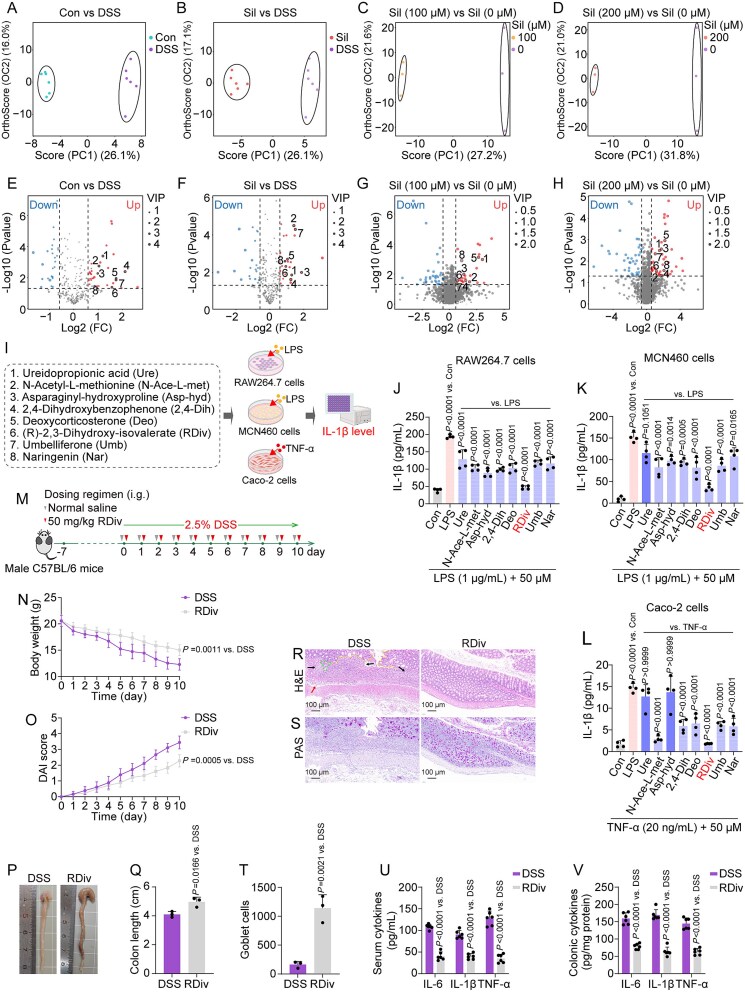
Metabolic profiles of silibinin-regulated fecal microbiota *in vivo* and *ex vivo*. (A–D) OPLS-DA plots for pairwise group comparisons of metabolic profiles. (E–H) volcano plots highlighting significantly altered metabolites in pairwise comparisons, indicating upregulated and downregulated metabolites. (I) Schematic representation of the construction, intervention, and pharmacodynamic characterization of the *in vitro* models of UC (elements created with BioRender.com; the graphical abstract shares the same source). (J–L) Measured levels of IL-1β in cell supernatant following treatments. (M) Schematic diagram illustrating the mouse experimental setup. (N, O) Dynamic curves of body weight and DAI scores over time. (P, Q) Phenotypic presentation of colons and colon length. (R, S) Representative H&E (arrows indicate inflammatory cell infiltration or mucosal swelling; dashed lines denote crypt distortion or irregularities on the crypt surface) and PAS staining micrographs. (T) Quantitative analysis of goblet cells in PAS staining. (U, V) Quantified levels of IL-6, IL-1β, and TNF-α in serum and colonic tissue. Data are presented as the mean ± SD (*n* = 3–6). *P*-values for each comparison were indicated.

Recognizing the significant impact of gut microbiota metabolites on host health and various disease states, we aimed to reveal the metabolic profile of gut microbiota regulated by silibinin. The metabolic profiles of gut microbiota were elucidated in both *in vivo* and *ex vivo* conditions through the application of untargeted LC–MS. Through MS/MS analysis, we identified a total of 318 metabolites in feces and 1775 metabolites in the *ex vivo* fecal microbiota. These metabolites were subsequently subjected to further analysis employing orthogonal projections to latent structures-discriminant analysis (OPLS-DA). The OPLS-DA plots revealed a significant separation of metabolic profiles between the control and DSS groups (Con versus DSS) (Fig. 4A). Furthermore, significant separations were observed between the silibinin treatment at 50 mg/kg and the DSS group (Sil versus DSS), as well as between the 100-μM silibinin and 0-μM silibinin groups [Sil (100 μM) versus Sil (0 μM)], and between the 200-μM silibinin and 0-μM silibinin groups [Sil (200 μM) versus Sil (0 μM)] (Fig. 4B–D). Differential metabolites of significance for each pairwise comparison were discerned according to specified criteria: a *P*-value of <.05, a fold change >1.5 or <0.8, and a variable importance in projection exceeding 1. Accordingly, volcano plots were employed to delineate the attributes of the significant differential metabolites across the comparisons of Con versus DSS, Sil versus DSS, Sil (100 μM) versus Sil (0 μM), and Sil (200 μM) versus Sil (0 μM), respectively (Fig. 4E–H). Eight microbiota-derived metabolites—ureidopropionic acid, N-acetyl-L-methionine, asparaginyl-hydroxyproline, 2,4-dihydroxybenzophenone, deoxycorticosterone, (R)-2,3-dihydroxy-isovalerate, umbelliferone, and naringenin—demonstrated significant alterations subsequent to DSS treatment. Nevertheless, a trend toward normalization was evident following the restoration of gut microbiota through silibinin intervention.

*In vitro* models of UC were established through the stimulation of RAW264.7 and NCM460 cells with LPS and Caco-2 cells with TNF-α to evaluate the anti-UC activities of the eight metabolites (Fig. 4I). Preliminary investigations indicated that stimulation with 1 μg/ml of LPS in RAW264.7 and NCM460 cells and 20 ng/ml of TNF-α in Caco-2 cells significantly enhanced the levels of IL-1β, with negligible cytotoxic effects observed. Consequently, 1 μg/ml of LPS and 20 ng/ml of TNF-α were chosen for further experimentation (data not shown). The majority of metabolites demonstrated minimal cytotoxicity when applied to LPS-stimulated RAW264.7 and NCM460 cells and TNF-α-stimulated Caco-2 cells at concentrations reaching 50 μM (Supplementary Figs S1–S3). Consequently, the concentration of the eight metabolites utilized in subsequent *in vitro* experiments was established at 50 μM. (R)-2,3-dihydroxy-isovalerate demonstrated the most potent inhibition of IL-1β production induced by LPS and TNF-α at equivalent concentrations (Fig. 4J–L), thereby underscoring its significant *in vitro* anti-UC activity. Next, we designed animal experiments to further investigate the role of (R)-2,3-dihydroxy-isovalerate in the anti-UC response (Fig. 4M). (R)-2,3-dihydroxy-isovalerate substantially mitigated various symptoms associated with DSS challenge, including reductions in body weight, increased DAI scores, shortened colon length, and histopathological damage and inflammation within the colon (Fig. 4N–V). Besides, the co-administration of (R)-2,3-dihydroxy-isovalerate and silibinin in ABX-induced pseudo-germ-free mice markedly renewed the protective effects of silibinin against DSS-induced body weight loss, progression of the DAI, reduction in colon length, inflammatory damage to the colon, and disruptions in intestinal immune function (Fig. 3B–N). In conclusion, (R)-2,3-dihydroxy-isovalerate potentially serves as a key contributor in the efficacy of silibinin-corrected gut microbiota in the treatment of UC.

**Figure 5:**
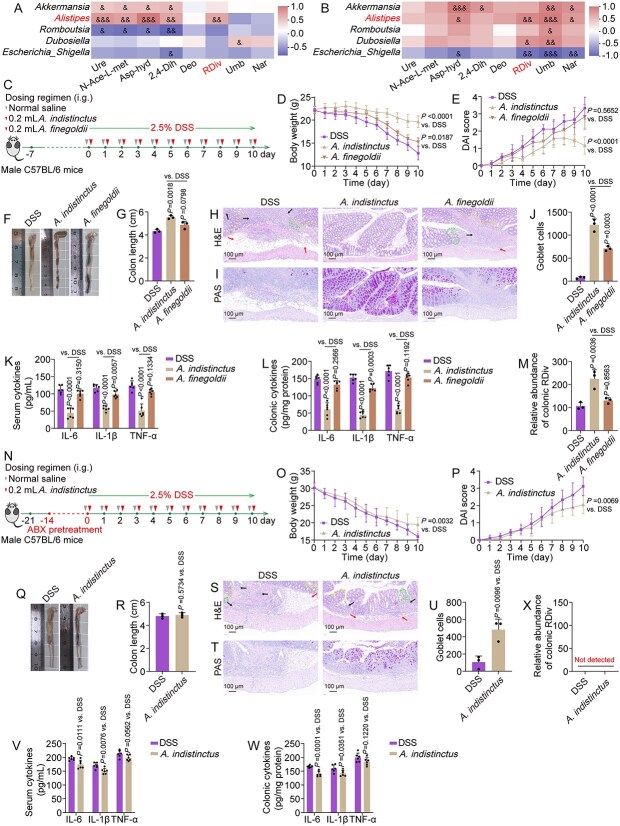
*Alistipes* may exert a positive effect on DSS-induced mice by indirectly producing (R)-2,3-dihydroxy-isovalerate. (A, B) Spearman correlation analysis between key bacterial taxa and differential metabolites. In Spearman correlation analysis, ^*^P < .05, ^**^P < .01, and ^***^P < .001. (C, N) workflow of the study design with or without ABX. (D, E, O, and P) Temporal dynamics of body weight and DAI scores. (F, G, Q, and R) Phenotypic observations of the colon and measurements of colon length. (H, I, S, and T) H&E (arrows indicate inflammatory cell infiltration or mucosal swelling; dashed lines denote crypt distortion or irregularities on the crypt surface) and PAS staining micrographs. (J, U) Quantitative analysis of goblet cells in PAS staining. (K, L, V, and W) levels of IL-6, IL-1β, and TNF-α in serum and colonic tissue. (M, X) detection of (R)-2,3-dihydroxy-isovalerate abundance in the colon. Data are presented as the mean ± SD (*n* = 3–6). *P*-values for each comparison were indicated.

A Spearman correlation analysis was performed to examine the relationship between the shared microbial biomarkers and the significantly differential metabolites. A positive correlation was observed between the abundance of *Alistipes* and the content of (R)-2,3-dihydroxy-isovalerate (Fig. 5A and B). We then selected the representative species *A. indistinctus* and *A. finegoldii* from the genus *Alistipes* to investigate the relationship between these bacteria and (R)-2,3-dihydroxy-isovalerate in the context of UC (Fig. 5C and N). *A. indistinctus* markedly mitigated weight loss, elevated DAI, colonic shortening, inflammatory damage, and goblet cell depletion in mice induced with DSS, whereas *A. finegoldii* exhibited a moderate ameliorative effect (Fig. 5D–L). Furthermore, intervention with *A. indistinctus* led to an increase in colonic (R)-2,3-dihydroxy-isovalerate levels, whereas *A. finegoldii* exhibited only a nonsignificant upward trend. (Fig. 5M). However, in ABX-induced pseudo-germ-free mice, the beneficial effects of *A. indistinctus* on mitigating DSS-induced decreases in body weight, elevated DAI scores, reduced colon length, and histopathological damage and inflammation were diminished (Fig. 5O–W). More importantly, *A. indistinctus* failed to enhance (R)-2,3-dihydroxy-isovalerate production in these mice (Fig. 5X). These findings suggest that although *Alistipes* does not directly synthesize (R)-2,3-dihydroxy-isovalerate, it may interact with other gut microbes to promote its accumulation, thereby contributing to the alleviation of UC.

### (R)-2,3-dihydroxy-isovalerate bound to the GAT-3 protein directly

**Figure 6:**
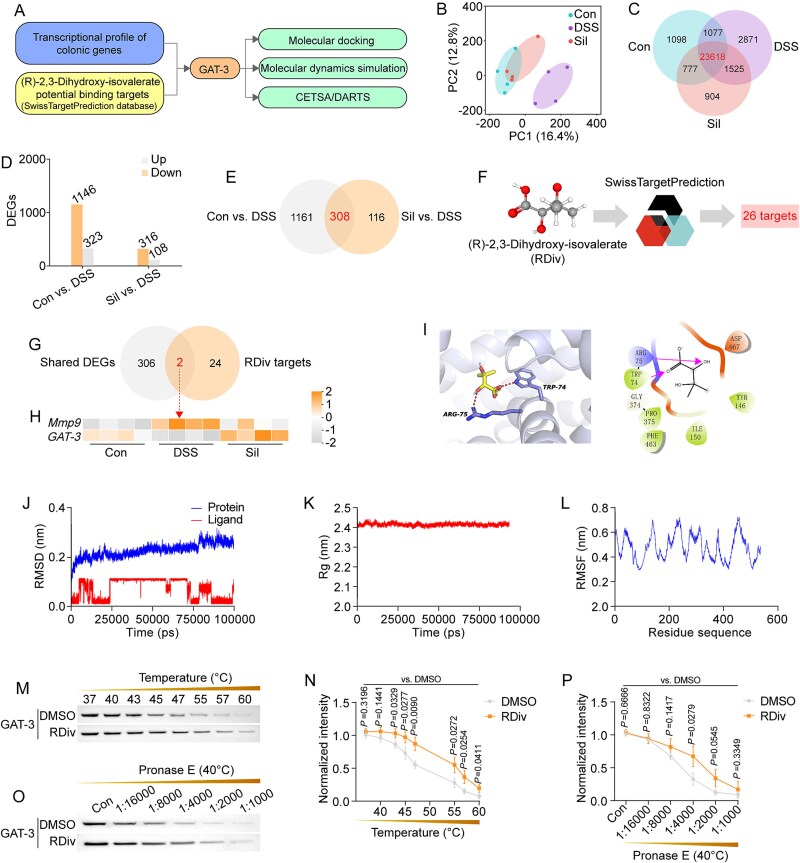
Identification and validation of the direct interaction between (R)-2,3-dihydroxy-isovalerate and GAT-3. (A) Workflow for identifying potential targets of (R)-2,3-dihydroxy-isovalerate. (B) PCA plot illustrating gene expression in each group. (C, E, and G) Venn diagrams of overlapping genes or pairwise comparisons between groups. (D) Numbers of up- and downregulated DEGs in pairwise comparisons. (F) Target prediction process for (R)-2,3-dihydroxy-isovalerate. (H) Heat maps presenting the expression of *MMP9* and *GAT-3* in the colon. (I) Molecular docking of (R)-2,3-dihydroxy-isovalerate with GAT-3. (J) Backbone root mean square deviation fluctuations of (R)-2,3-dihydroxy-isovalerate and GAT-3. (K, L) Fluctuations in Rg and root mean square fluctuation values during molecular dynamics simulations. (M, N) CETSA with corresponding semiquantitative analysis of GAT-3 thermal stabilization in the presence of (R)-2,3-dihydroxy-isovalerate. (O, P) DARTS with semiquantitative analysis of enzyme stabilization of GAT-3 by (R)-2,3-dihydroxy-isovalerate. Data are presented as the mean ± SD (*n* = 3–4). *P*-values for each comparison were indicated.

To identify the potential targets of (R)-2,3-dihydroxy-isovalerate for the mitigation of DSS-induced UC, we employed methodologies rooted in transcriptomics, computational biology, and systems pharmacology (Fig. 6A). First, transcriptomic analysis of the colon tissue from mice subjected to silibinin intervention revealed significant insights. The results of PCA demonstrated strong internal consistency across all treatment groups (Fig. 6B). Silibinin intervention was effective in normalizing gene expression profiles in the colons of these mice, aligning them more closely with those observed in healthy controls. A total of 23 618 genes were found to be shared among the gene expression profiles of the various treatment groups (Fig. 6C), and these genes were subsequently analyzed in greater detail based on specific criteria: an adjusted *P*-value of <.01 and a fold change exceeding 2 or falling below 0.5. Differentially expressed genes (DEGs) were identified by comparing the control group to the DSS group (Con versus DSS), revealing a total of 323 genes that were upregulated and 1146 genes that were downregulated (Fig. 6D). In the silibinin-treated group, a comparison with the DSS group (Sil versus DSS) indicated that 108 genes were upregulated, whereas 316 genes exhibited downregulation. In these two comparisons, we identified 308 shared DEGs (Fig. 6E). By comparing these 308 DEGs with the 26 potential drug targets of (R)-2,3-dihydroxy-isovalerate revealed in the SwissTargetPrediction database (Fig. 6F), we identified *matrix metallopeptidase 9* (*MMP9*) and *GAT-3* as the convergent drug targets of (R)-2,3-dihydroxy-isovalerate (Fig. 6G, H). Preliminary theoretical studies indicated that the binding affinity of (R)-2,3-dihydroxy-isovalerate to MMP-9 surpassed that of (R)-2,3-dihydroxy-isovalerate to GAT-3 (Supplementary Fig. S4). Moreover, numerous studies have demonstrated that MMP9 is primarily involved in the migration and invasion of tumor cells [20, 21]. Conversely, GAT-3 is an integral element of the GABAergic signaling pathway, significantly contributing to the modulation of immune signaling within the body [22]. Given that the gut represents the largest immune organ in human, our research focus has been directed toward GAT-3.

We proved that the GAT-3 protein represents a crucial target through which (R)-2,3-dihydroxy-isovalerate mediates its effects, thereby enhancing the anti-UC properties of silibinin-regulated gut microbiota. (R)-2,3-dihydroxy-isovalerate occupied the binding pocket of GAT-3, engaging in interactions with residues Arg75 and Trp74 (Fig. 6I). Next, the conformational stability of the (R)-2,3-dihydroxy-isovalerate-GAT-3 complex was further assessed using molecular dynamics simulation. The root mean square deviation of the complex system attained equilibrium during the final 20 ns, and fluctuations remain below 2.0 Å (Fig. 6J), thereby signifying the stability of the protein–ligand interaction. The radius of gyration (Rg) values, which consistently hovered around 2.4 nm (Fig. 6K), corroborated the stability and compactness of the complex. Furthermore, the root mean square fluctuation of the amino acid residues within the binding pocket exhibited only minor fluctuations (Fig. 6L), indicating a stable conformation of the binding pocket throughout the molecular dynamic simulations. Results from the cellular thermal shift assay (CETSA) analysis revealed a significantly elevated thermal stability of GAT-3 in the (R)-2,3-dihydroxy-isovalerate treatment group compared to the untreated group (Fig. 6M and N). This finding suggests that (R)-2,3-dihydroxy-isovalerate has enhanced the thermal stability of GAT-3. Moreover, findings from the drug affinity responsive target stability (DARTS) experiment indicated that (R)-2,3-dihydroxy-isovalerate effectively mitigated the pronase E-induced degradation of GAT-3 (Fig. 6O and P), suggesting an enhancement in the enzymatic stability of this protein. Together, (R)-2,3-dihydroxy-isovalerate demonstrated a direct binding interaction with GAT-3, which is the key target involved in its regulation of UC.

### (R)-2,3-dihydroxy-isovalerate-mediated GAT-3/RARβ/RORγt axis primarily contributed to the effects on DSS-induced mice

**Figure 7:**
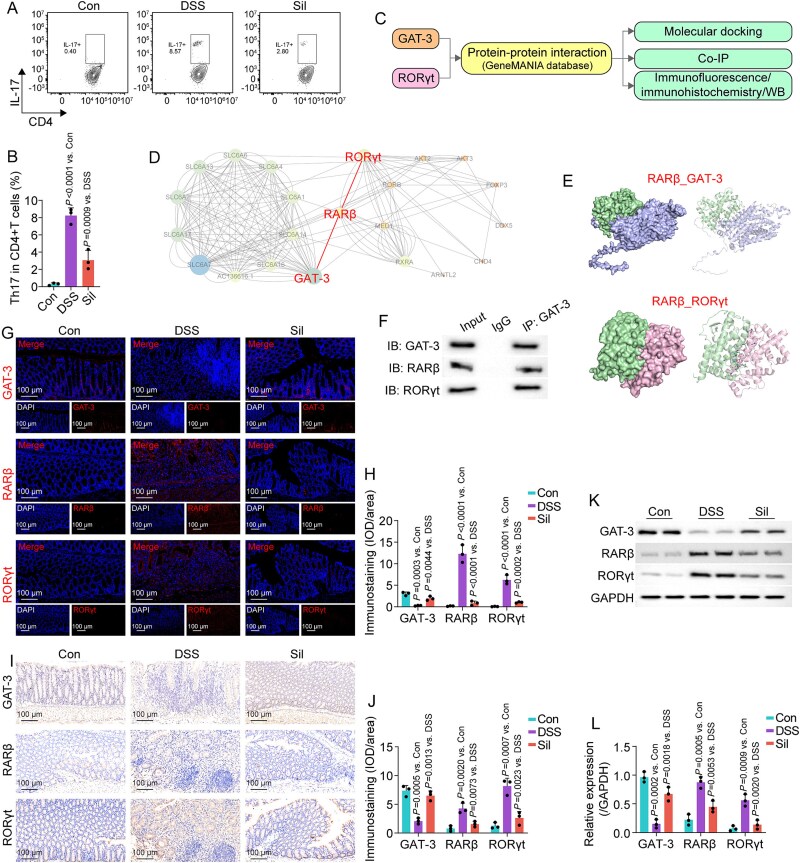
GAT-3/RARβ/RORγt axis may be involved in the benefits of silibinin on DSS-induced mice. (A, B) characterization and quantitative analysis of colonic Th17 cell infiltration. (C) Workflow for identifying the protein–protein interaction (PPI) between GAT-3 and RORγt. (D) PPI network analysis of GAT-3, RARβ, and RORγt. (E) Molecular docking simulations of RARβ with GAT-3 and RARβ with RORγt. (F) Co-IP assay demonstrating the interaction between GAT-3 and RARβ, RORγt in colon tissue. (G, H) representative fluorescent micrographs and corresponding quantification of GAT-3, RARβ, and RORγt in colonic tissue. (I, J) Representative immunohistochemical micrographs and quantitative analysis of GAT-3, RARβ, and RORγt expression in colonic sections. (K, L) Expression levels and semiquantitative analysis of GAT-3, RARβ, and RORγt in colonic tissues. Data are presented as the mean ± SD (*n* = 3). *P*-values for each comparison were indicated.

Recent research has identified IL-17-producing T cells as pivotal contributors to the pathogenesis of UC [23]. Furthermore, an in-depth analysis of the Gene Expression Omnibus (GEO) database (GSE59071) indicates that patients with active UC demonstrate significantly elevated expression levels of RORγt—a critical regulator of Th17 cell differentiation—in the intestinal mucosa, in comparison to individuals with inactive UC (Supplementary Fig. S5). Consistent with this observation, a significant increase in Th17 cell infiltration was noted in DSS-challenged mice (Fig. 7A and B). Our study demonstrated that silibinin treatment effectively suppressed this infiltration. Following the depletion of gut microbiota, the inhibitory effect of silibinin on Th17 cell differentiation was reduced (Supplementary Fig. S6). However, both FMT and (R)-2,3-dihydroxy-isovalerate were able to restore silibinin’s inhibitory effect on Th17 cell differentiation. Consequently, silibinin appeared to restore the gut microbiota, thereby inhibiting Th17 cell differentiation. We then hypothesize that the combination of (R)-2,3-dihydroxy-isovalerate and GAT-3 may modulate the silibinin-regulated gut microbiota to enhance the Th17 response in the colons of mice.

**Figure 8:**
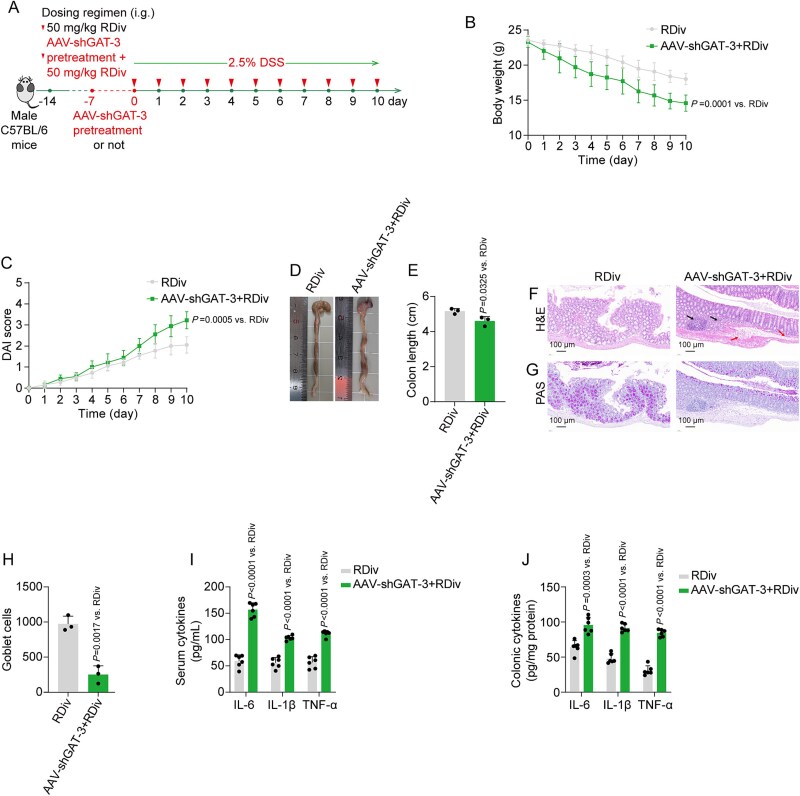
AAV-shGAT-3 abolished the therapeutical effects of (R)-2,3-dihydroxy-isovalerate on DSS-induced mice. (A) Schematic diagram of the *in vivo* study design. (B, C) Dynamic curves of body weight and DAI scores. (D, E) Colon phenotype and length measurements. (F, G) Representative H&E (arrows indicate inflammatory cell infiltration or mucosal swelling; dashed lines indicate irregularities on the crypt surface) and PAS staining micrographs of colon tissue. (H) Quantitative analysis of goblet cells in PAS staining. (I, J) quantified levels of IL-6, IL-1β, and TNF-α in serum and colonic tissue. Data are presented as the mean ± SD (*n* = 3–6). *P*-values for each comparison were indicated.

RORγt was initially predicted to interact with GAT-3 using the GeneMANIA database and subsequently validated through a series of comprehensive experiments (Fig. 7C). The GeneMANIA database predicts that GAT-3 is likely to interact with RORγt predominantly via RARβ (Fig. 7D). GAT-3 demonstrated an affinity for binding RARβ, which in turn displayed a binding interaction with RORγt (Fig. 7E; Supplementary Fig. S7). In addition, the interaction between these proteins was validated using co-immunoprecipitation (co-IP) assays (Fig. 7F). Immunohistochemical analyses lend substantial support to the notion that the (R)-2,3-dihydroxy-isovalerate-related GAT-3/RARβ/RORγt axis plays a critical role in the biological effects of silibinin. In the DSS group, a discernibly low staining intensity (brown/fluorescence) for GAT-3 was noted, whereas RARβ and RORγt exhibited robust staining intensity, suggesting mussy levels of these proteins (Fig. 7G–J). In contrast, the alteration in staining intensity was significantly ameliorated following silibinin treatment. In line with the above results, the DSS group exhibited a significant reduction in the protein levels of GAT-3, alongside a marked increase in the levels of RARβ and RORγt, when compared to the control group (Fig. 7K and L). Supplemented silibinin effectively reversed these changes. In summary, the (R)-2,3-dihydroxy-isovalerate-mediated GAT-3/RARβ/RORγt axis may be involved in the anti-UC properties of silibinin-regulated gut microbiota.

**Figure 9:**
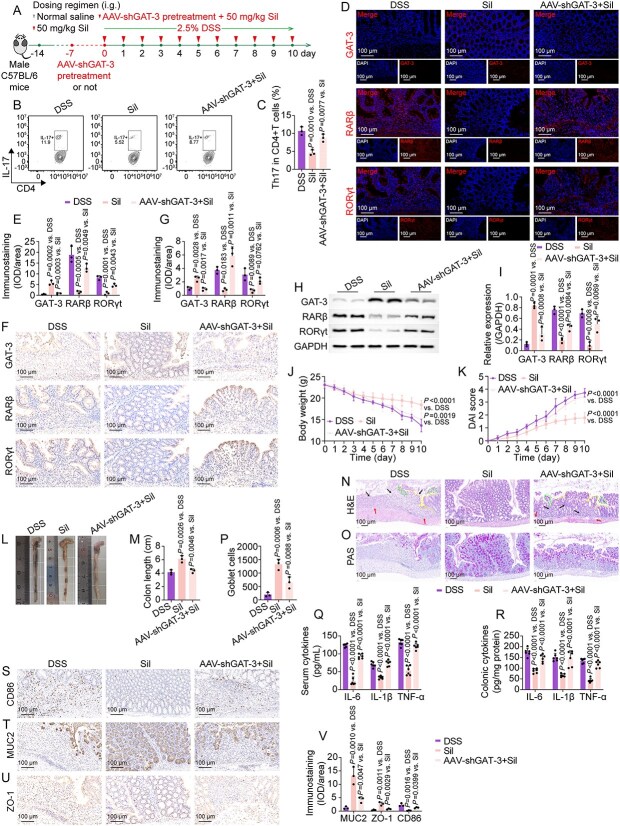
AAV-shGAT-3 abolished the silibinin-mediated protective effects on DSS-induced mice through GAT-3/RARβ/RORγt axis. (A) Schematic overview of the experimental design. (B, C) Characterization and quantitative analysis of colonic Th17 cell infiltration. (D, E) Representative fluorescent micrographs and quantitative analysis of GAT-3, RARβ, and RORγt. (F, G) Representative immunohistochemical micrographs and quantitative analysis of GAT-3, RARβ, and RORγt. (H, I) Expressions and semiquantitative analysis of GAT-3, RARβ, and RORγt. (J, K) Dynamic curves of body weight and DAI scores. (L, M) Phenotypic presentation of colons and colon length. (N, O) Representative micrographs of H&E (arrows indicate inflammatory cell infiltration or mucosal swelling; dashed lines denote crypt distortion or irregularities on the crypt surface) and PAS staining. (P) Quantitative analysis of goblet cells in PAS staining. (Q, R) The levels of IL-6, IL-1β, and TNF-α in the serum and colon. (S–V) Representative immunohistochemical images and quantitative analysis of the staining intensity of CD86, MUC2, and ZO-1 in the colon sections. Data are presented as the mean ± SD (*n* = 3–6). *P*-values for each comparison were indicated.

To confirm whether the anti-UC effects were associated with the initiation of the GAT-3/RARβ/RORγt axis, we employed stereotactic delivery of an AAV9 virus encoding shRNA sequences against the GAT-3 gene (AAV-shGAT-3) directly into the colons of mice (Figs 8A and 9A). One week following viral delivery, the colonic tissue from AAV-shGAT-3-treated mice exhibited reduced GAT-3 expression relative to that observed in untreated mice or those receiving the scramble vector (AAV-shCon) (Supplementary Fig. S8). The knockdown of GAT-3 eliminated the therapeutic effects of (R)-2,3-dihydroxy-isovalerate on DSS-induced mice, as evidenced by its inability to mitigate body weight loss, reduce DAI scores, prevent colon shortening, and ameliorate inflammatory lesions in the colon (Fig. 8B–J). Furthermore, the inhibitory effects of silibinin on Th17 cell infiltration, as well as on the protein expression of GAT-3-modulated RARβ and RORγt, were completely abrogated following the knockdown of colonic GAT-3 (Fig. 9B–I). The beneficial effects of silibinin on mitigating body weight loss, disease progression, colon length shortening, colonic inflammatory infiltration, and immune abnormalities were markedly diminished when co-treatment with GAT-3 knockdown was administered (Fig. 9J–V). These findings collectively underscore the role of (R)-2,3-dihydroxy-isovalerate in mediating the therapeutic effects of silibinin-modulated gut microbiota on DSS-induced colitis, specifically through the GAT-3/RARβ/RORγt signaling axis.

## Discussion

UC represents a major health concern due to its high prevalence and considerable disease severity, highlighting the urgent need for the development of effective therapeutic interventions [3]. The modulation of gut microbiota presents itself as a promising and safe alternative treatment strategy for UC [3]. Silibinin, a commonly used dietary supplement, is acknowledged for its significant medicinal and nutritional attributes [11]. Furthermore, the modulation of gut microbiota has been identified as a mechanism contributing to the efficacy of silibinin. In this study, we report that silibinin ameliorated gut microbiota composition and enhanced the production of microbiota-derived (R)-2,3-dihydroxy-isovalerate. This metabolite conferred protection to mice against DSS-induced UC through the GAT-3/RARβ/RORγt axis. Given the well-established gut–liver axis, silibinin’s influence on gut microbial homeostasis may exert effects that extend beyond intestinal health [24]. Emerging evidence indicates that its therapeutic efficacy in MASLD is largely mediated through modulation of the gut microbiota, which in turn confers benefits via the gut–liver axis [24]. Thus, silibinin-induced alterations in gut microbial communities may represent a key mechanism underlying its hepatoprotective effects. Further clinical studies are warranted to explore the dual benefits of silibinin-mediated microbiota modulation in both hepatic and intestinal disorders, particularly in patients with coexisting colitis and liver dysfunction.

Pharmacokinetic studies have demonstrated that silibinin’s oral bioavailability in rats is merely 0.73% [11], indicating that a significant portion of unabsorbed silibinin remains within the intestinal lumen. It is well established that the intestine harbors a substantial population of gut microbiota, which functions as a critical “organ” in the pathogenesis and progression of UC [5]. In our study, we demonstrated that oral administration of silibinin effectively shifted DSS-induced dysbiosis of gut microbiota both *in vivo* and *ex vivo*, as indicated by the restoration of microbial diversity and community composition. The anti-UC efficacy of silibinin was markedly diminished in pseudo-germ-free mice, yet these effects were rescued through FMT from silibinin-treated mice or metabolite produced by silibinin-mediated gut microbiota. These findings collectively highlight the pivotal role of interactions between unabsorbed silibinin and gut microbiota in mediating the therapeutic effects on DSS-induced colitis. In detail, the anti-UC effects of silibinin may be attributed to significant alterations in the relative abundance of microbial biomarkers, including *Alistipes*, *Romboutsia*, *Escherichia-Shigella*, *Akkermansia*, and *Dubosiella*. Clinical studies have revealed a pronounced increase in the abundance of *Escherichia-Shigella* within the inflamed mucosa of patients suffering from active UC [25]. Moreover, elevated levels of both *Romboutsia* and *Escherichia-Shigella* have been observed in DSS-induced mice [25, 26]. Conversely, *Akkermansia* and *Dubosiella* have been noted for their probiotic-like properties, which help mitigate intestinal inflammation through various mechanisms [27, 28]. *Alistipes* is frequently linked to chronic intestinal inflammation; however, recent studies suggest that it may also play a protective role against UC under certain conditions [29]. Herein, we found that *A. indistinctus* and *A. finegoldii* mitigated various symptoms induced by DSS to differing extents, thereby reinforcing the probiotic-like function of *Alistipes* in UC. Depletion of the gut microbiota abolished the protective effects of *A. indistinctus* against UC, suggesting that the beneficial impact of *Alistipes* is dependent on its interactions with other gut microbial communities.

It has been estimated that ~36% of small-molecule compounds present in human blood are synthesized or modified through microbial metabolism [30]. Meanwhile, comparisons of blood metabolites between germ-free and wild-type mice indicate that hundreds of metabolites are uniquely identified in wild-type mice [31]. In our prior study, dietary supplementation with *Chlorella pyrenoidosa* enhanced gut microbiota-derived butyrate production, which consequently inhibited colonic cell apoptosis and mitigated DSS-induced UC [3]. Therefore, we propose that silibinin interacts with the gut microbiota, resulting in alterations to their metabolic profiles that are crucial in mediating the anti-UC effects associated with silibinin-modulated gut microbiota. Herein, our metabolomic analysis, conducted both *in vivo* and *ex vivo*, revealed that silibinin-modulated gut microbiota exhibited a distinct metabolic profile. Notably, eight microbially derived metabolites were identified as key contributors within this metabolic framework. Among these metabolites, (R)-2,3-dihydroxy-isovalerate, a dihydroxy derivative with a structure similar to SCFAs, was identified. However, its biological activity has not been previously reported. There is substantial evidence supporting the use of LPS-induced RAW264.7 and NCM460 cell models, as well as TNF-α-stimulated Caco-2 cells, as effective *in vitro* systems for elucidating the anti-inflammatory mechanisms of pharmacological agents in UC [32, 33]. Among the metabolites identified, (R)-2,3-dihydroxy-isovalerate exhibited the most significant anti-inflammatory effects *in vitro* and demonstrated protective effects *in vivo*. Moreover, (R)-2,3-dihydroxy-isovalerate was found to restore the protective effects of silibinin against DSS-induced colitis symptoms in pseudo-germ-free mice. Thus, the silibinin-mediated gut microbiota/(R)-2,3-dihydroxy-isovalerate pathway emerges as a crucial mechanism underlying the therapeutic effects of gut microbiota regulation on DSS-induced colitis. Further analysis demonstrated a positive correlation between *Alistipes* and (R)-2,3-dihydroxy-isovalerate. Recognized as a potential SCFA producer, *Alistipes* has been shown to generate acetate and propionate within the mammalian gastrointestinal tract [34]. In this study, oral administration of *A. indistinctus* and *A. finegoldii* increased the colonic abundance of (R)-2,3-dihydroxy-isovalerate. This effect was notably reduced in pseudo-germ-free mice, indicating that *Alistipes* may indirectly promote the production of this compound through interactions with other gut microbiota. (R)-2,3-dihydroxy-isovalerate is a metabolite that plays a role in the synthesis pathway of branched-chain amino acids, which is mediated by gut microbiota. However, the exact mechanisms underlying the synthesis and metabolism of (R)-2,3-dihydroxy-isovalerate by gut bacteria have yet to be fully elucidated. Furthermore, it remains to be determined whether other species within the *Alistipes* genus are capable of directly producing (R)-2,3-dihydroxy-isovalerate.

Further analysis identified GAT-3 as the common target between the colonic transcriptome and the predicted targets of (R)-2,3-dihydroxy-isovalerate. To investigate this relationship in greater depth, we demonstrated that (R)-2,3-dihydroxy-isovalerate exhibits a direct and stable interaction with GAT-3 through the application of molecular docking, molecular dynamics simulations, CETSA, and DARTS assays. Furthermore, in DSS-induced UC mice, the expression of colonic GAT-3 was significantly downregulated, but was notably restored following treatment with silibinin. Collectively, our findings confirm that GAT-3 acts as a downstream component within the silibinin-mediated gut microbiota/(R)-2,3-dihydroxy-isovalerate pathway. GAT-3, a member of the solute carrier superfamily 6, operates as a sodium-dependent neurotransmitter transporter and is potentially pivotal in cellular electrophysiology, as well as in the modulation of gastrointestinal motility and sensory neuron activity [35]. However, there is a paucity of research directly connecting GAT-3 to UC. In mammals, the enteric nervous system (ENS) is situated within the myenteric and submucosal plexuses and comprises enteric neurons and glial cells [22, 36]. The ENS is intricately associated with both adaptive and innate immune cells throughout the gastrointestinal tract, enabling interactions that are crucial for mediating intestinal immunity and tissue repair [36]. Studies have indicated that the ENS possesses distinctive immunosuppressive properties in UC [37]. Reportedly, GAT-3 expression has been observed in the ENS [38], implying a potential involvement of GAT-3 in ENS-mediated intestinal immunity. There is a growing scholarly interest in the interaction between GATs and the intestinal immune system [39–42]. Reportedly, a deficiency in the GAT family member GAT-2 led to the differentiation of intestinal germinal center B cells and exacerbated immunoglobulin A nephropathy symptoms in a mouse model [41]. A study demonstrated that the absence of GAT-2 heightened the Th17 response in mouse models of intestinal infection and inflammation [42]. It has been documented that GAT-1 deficiency leads to increased phosphorylation of IκB kinase and activation of the T-bet-STAT1 signaling pathway, which in turn facilitates the differentiation of Th1 and Th17 cells [43]. Furthermore, research has shown that enteric neurons modulate Th17 cell differentiation through various mechanisms [44]. Given the expression of GATs by the ENS [38], these findings further substantiate the involvement of GATs in the regulation of Th17 cells. Thus, we hypothesize that GAT-3 may influence the progression of UC by affecting the intestinal immune system, with a specific focus on the differentiation of Th17 cells.

Th17 cells, characterized by RORγt-dependent production of IL-17, constitute a critical subset of CD4^+^ T cells within the intestine [45]. These cells have the potential to assume a pathogenic role and are positively associated with various chronic inflammatory diseases, including multiple sclerosis, rheumatoid arthritis, psoriasis, and IBD [45]. Moreover, the differentiation of Th17 cells and the upregulation of RORγt expression in the colonic tissue are commonly observed in UC patients [45]. In our study, mice with DSS-induced UC demonstrated infiltration of Th17 cells, aligning with findings from a GEO clinical study. Nevertheless, the excessive infiltration of Th17 cells was mitigated by modulating the gut microbiota via silibinin, suggesting that the regulation of Th17 cell differentiation is a crucial step in the silibinin-mediated gut microbiota/(R)-2,3-dihydroxy-isovalerate pathway for alleviating UC. Reportedly, the *Alistipes*, which is positively associated with the biosynthesis of (R)-2,3-dihydroxy-isovalerate, contributed to the suppression of intestinal Th17 differentiation [46]. Prior studies have suggested that intestinal epithelial RARβ facilitates the differentiation of intestinal Th17 cells and enhances the production of IL-17 [47]. Consequently, the targeted inhibition of RARβ expression may serve as a promising therapeutic approach for modulating intestinal Th17 cell-mediated immunity in the context of infection or inflammation [47]. In this study, predicted protein interactions among GAT-3, RARβ, and RORγt were confirmed through co-IP. These findings imply that GAT-3 may indirectly influence the differentiation of intestinal Th17 cells via RARβ. In the colons of DSS-induced UC mice, both RARβ and RORγt were markedly upregulated, whereas GAT-3 expression was significantly downregulated. Conversely, the silibinin intervention effectively reversed these alterations. Moreover, pretreatment with AAV-shGAT-3, which successfully reduced GAT-3 expression in the colonic tissue of mice, negated all beneficial effects mediated by silibinin and (R)-2,3-dihydroxy-isovalerate on DSS-induced mice. These findings confirm that the (R)-2,3-dihydroxy-isovalerate-mediated GAT-3/RARβ/RORγt axis conferred the anti-UC effects of silibinin-regulated gut microbiota. Herein, silibinin enhanced GAT-3 expression, may through increasing its protein stability, supporting our hypothesis that GATs inhibit Th17 differentiation. The followed downregulation of RARβ and RORγt expression was consistent with previously reported patterns in the differentiation of Th17 cells within the intestine [45, 47]. Nevertheless, the present findings do not clarify the mechanism by which increased GAT-3 expression is associated with decreased RARβ and RORγt expression. Moreover, there is a lack of literature addressing the interactions among GAT-3, RARβ, and RORγt. Consequently, comprehensive experimental studies, such as cycloheximide chase assay and ubiquitination assay, are required to elucidate the regulatory relationships among these three proteins.

In the pharmacological evaluation, the administration of silibinin at a dosage of 100 mg/kg exhibited a reduced effect, aligning with our observations in MASLD mice and indicating that the action of silibinin is not dose-dependent. The low bioavailability of silibinin may result in metabolic saturation at lower doses [11], potentially accounting for its decreased efficacy at higher doses. The significant retention of high-dose silibinin within the intestines could adversely affect the gut microbiota, thereby diminishing the production of (R)-2,3-dihydroxy-isovalerate and, consequently, its effectiveness against UC. Furthermore, studies have suggested that silibinin possesses neuroregulatory properties [48], which raises the possibility that high doses may interact with the ENS in unanticipated ways and thereby influence the (R)-2,3-dihydroxy-isovalerate/GAT-3 pathway. In forthcoming research, we intend to design experiments to investigate the mechanisms underlying the differential pharmacological effects of silibinin at various dosages. Additionally, we aim to continue the collection and cultivation of other species within the genus *Alistipes* to further elucidate the relationship between (R)-2,3-dihydroxy-isovalerate and this genus. Utilizing GAT-3 knockout mice and cell lines, alongside various molecular biology techniques and high-throughput sequencing, we will conduct a comprehensive exploration of the connections among (R)-2,3-dihydroxy-isovalerate, GAT-3, and UC, as well as the interactions among GAT-3, RARβ, and RORγt. Furthermore, we plan to undertake clinical studies to collect samples from UC patients treated with silibinin to validate our findings.

## Conclusion

Our work identifies silibinin, an herbal monomer, as a regulator of gut microbiota, capable of restructuring the microbial environment to counteract UC-related disturbances, such as significant body weight loss, notable shortening of colon length, extensive colonic histopathological alterations with accompanying inflammation, and disruption of intestinal immune homeostasis. The therapeutic efficacy is closely linked to the targeting of the GAT-3/RARβ/RORγt axis through the enrichment of gut microbiota-derived (R)-2,3-dihydroxy-isovalerate. These findings enhance the limited understanding of the GAT-3/RARβ/RORγt axis and suggest that the properties of silibinin hold potential therapeutic value for UC treatment.

## Supplementary Material

Revised_supplementary_material_8_10_wraf175

## Data Availability

The sequence data generated in this study have been deposited in the SRA database under the accession code PRJNA1094477. The untargeted LC–MS data generated in this study have been archived in the MetaboLights database with the identifier MTBLS12546. The remaining data supporting the study’s conclusions will be available on Figshare at DOI: https://doi.org/10.6084/m9.figshare.29500703.
