# Supplementary material for: Silibinin-derived microbiota enrich (R)-2,3-dihydroxy-isovalerate and ameliorate colitis via the GAT-3/RARβ/RORγt axis
Source: ISME J. 2025 Aug 13;19(1):wraf175. doi: 10.1093/ismejo/wraf175 (PMC12448484; doi:10.1093/ismejo/wraf175)
Supplement: Revised_supplementary_material_8_10_wraf175 [file revised_supplementary_material_8_10_wraf175.docx]

**Supplementary material**

**Silibinin-drived microbiota enrich** **(R)-2,3-dihydroxy-isovalerate and** **ameliorate colitis via the GAT-3/RARβ/RORγt axis**

Baofei Yan^1†^, Xian Zheng^2†^, Danya Lu^1^, Ting Li^1^, Xi Chen^3^, Zhitao Shao^1^, Tingming Fu^1*^

^1^State Key Laboratory on Technologies for Chinese Medicine Pharmaceutical Process Control and Intelligent Manufacture, Nanjing University of Chinese Medicine, Nanjing 210023, P. R. China

^2^Department of Pharmacy, Affiliated Kunshan Hospital of Jiangsu University, Kunshan 215399, P. R. China

^3^Jiangsu Engineering Research Center for Precision Prevention and Treatment of Digestive and Reproductive System Tumors, Huaian 223003, P. R. China

^*^Corresponding authors: Tingming Fu, State Key Laboratory on Technologies for Chinese Medicine Pharmaceutical Process Control and Intelligent Manufacture, Nanjing University of Chinese Medicine, No. 138, Xianlin Avenue, Nanjing 210023, China. Email: futm@njucm.edu.cn

^†^These authors contributed equally to this work.

**Supplementary materials and methods**

**Detection of colonic** **(R)-2,3-dihydroxy-isovalerate**

Chromatographic separation was performed using an ACQUITY BEH C18 column (2.1 × 100 mm, 1.7 μm; Waters, MA, USA). The mobile phase comprised an aqueous solution of 0.1% formic acid (A) and 0.1% formic acid in acetonitrile (B). The elution gradient was programmed as follows: 0-3 min, 10% B; 3-10 min, 10%-35% B; 10-12 min, 35%-95% B; 12-14 min, 95% B; 14-14.5 min, 95%-10% B; and 14.5-16 min, 10% B. The flow rate was maintained at 0.3 mL/min, and the column temperature was held constant at 50℃. An electrospray ionization (ESI) source was employed, with an ionspray voltage of 5.5 kV, a temperature setting of 550℃, a curtain gas flow rate of 35 arb, a collision gas flow rate of 8 arb, and an ion source gas flow rate of 55 arb. The analysis was performed utilizing the multiple reaction monitoring (MRM) mode, employing a precursor ion with a mass-to-charge ratio (m/z) of 135.1 and a product ion with an m/z of 71.0. The declustering potential (DP) was configured at 83.00 V, while the collision energy (CE) was maintained at 10.88 V.


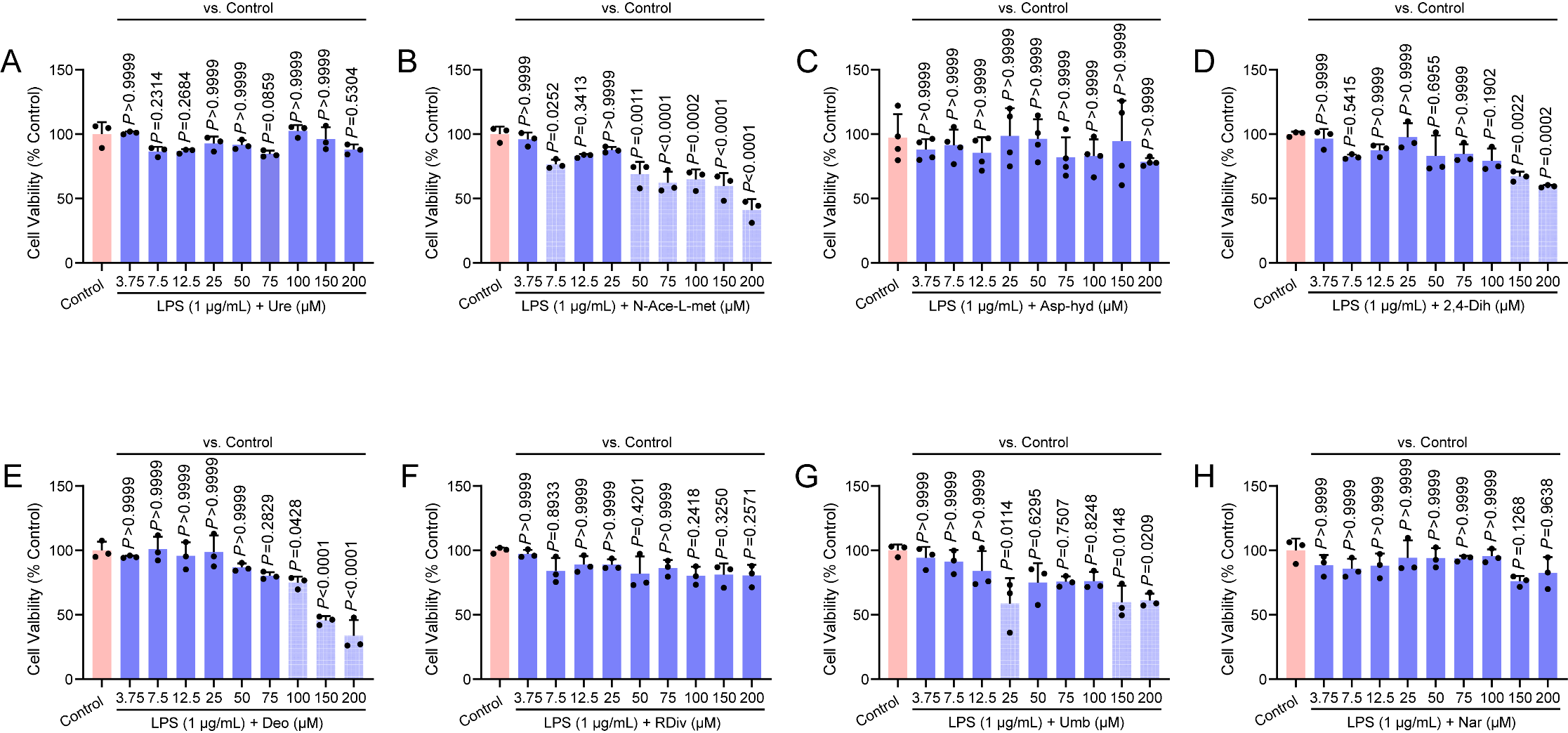


**Supplementary Figure 1.** The viability of LPS-induced RAW264.7 cells (A-H) was assessed following coculture with a series of concentrations of Ureidopropionic acid, N-Acetyl-L-methionine, Asparaginyl-Hydroxyproline, 2,4-Dihydroxybenzophenone, Deoxycorticosterone, (R)-2,3-Dihydroxy-Isovalerate, Umbelliferone, and Naringenin, respectively. Data are presented as the mean ±SD (n = 3). *P* values for each comparison were indicated.


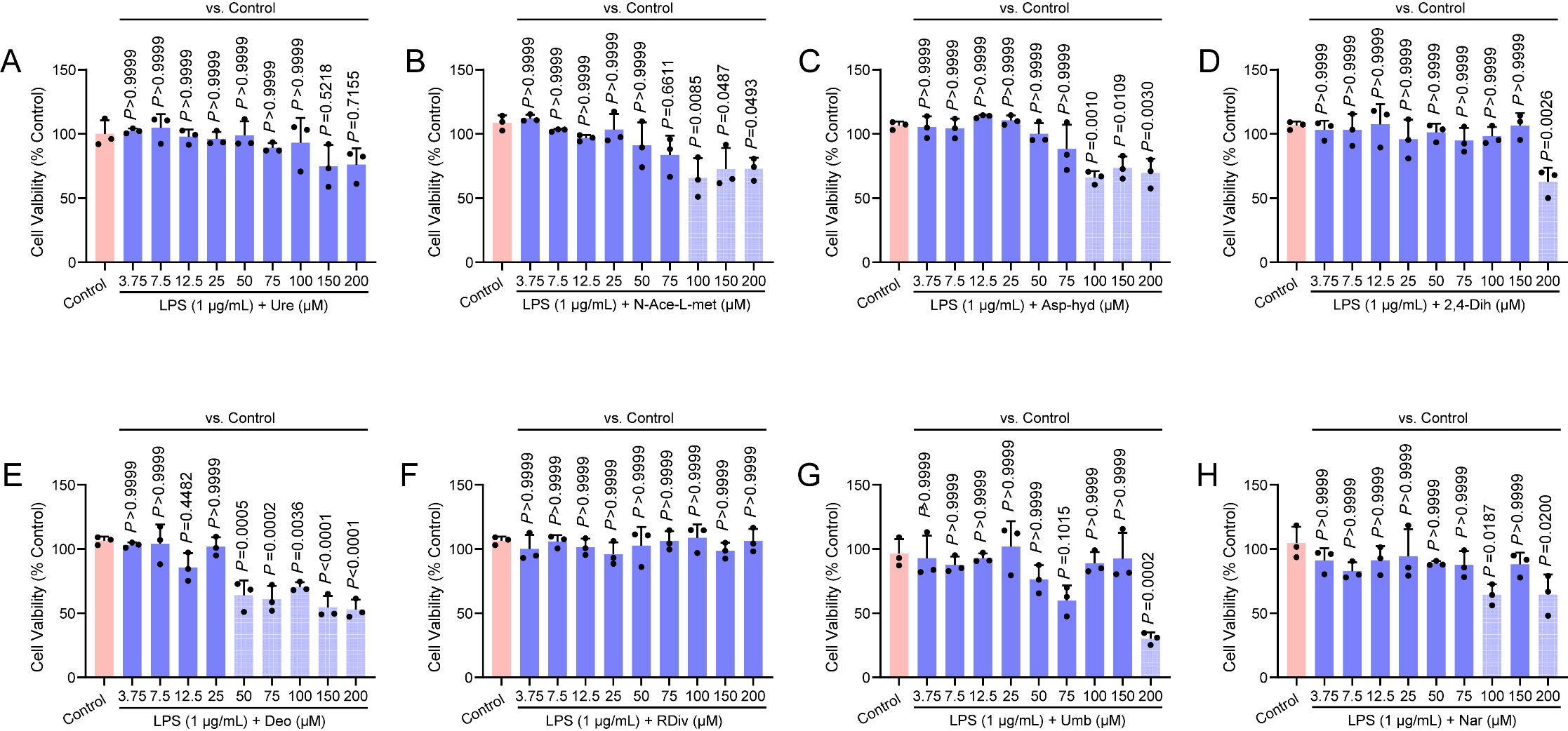


**Supplementary Figure 2.** The viability of LPS-induced NCM460 cells (A-H) was assessed following coculture with a series of concentrations of Ureidopropionic acid, N-Acetyl-L-methionine, Asparaginyl-Hydroxyproline, 2,4-Dihydroxybenzophenone, Deoxycorticosterone, (R)-2,3-Dihydroxy-Isovalerate, Umbelliferone, and Naringenin, respectively. Data are presented as the mean ±SD (n = 3). *P* values for each comparison were indicated.


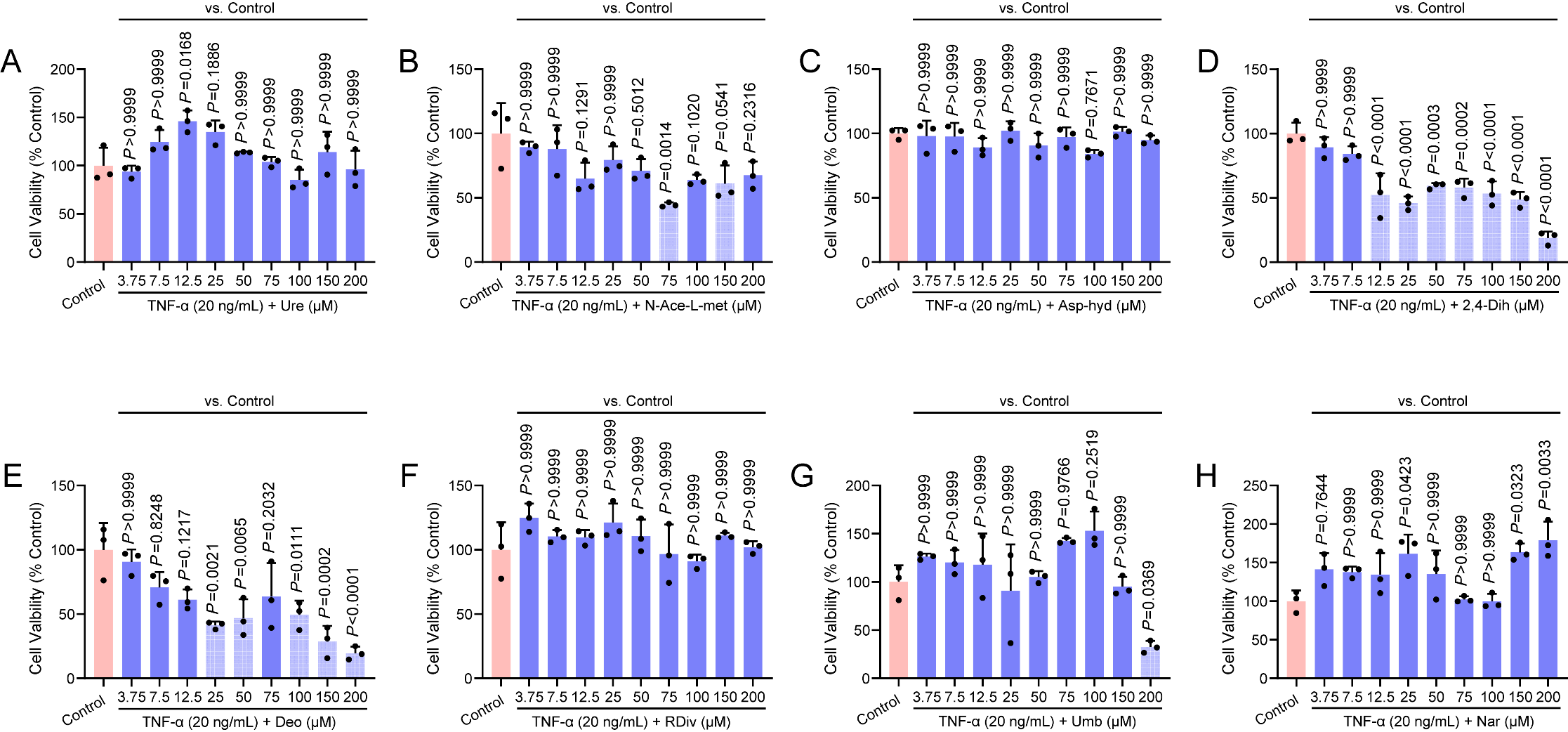


**Supplementary Figure 3.** The viability of TNF-α-stimulated Caco-2 cells (A-H) was assessed following coculture with a series of concentrations of Ureidopropionic acid, N-Acetyl-L-methionine, Asparaginyl-Hydroxyproline, 2,4-Dihydroxybenzophenone, Deoxycorticosterone, (R)-2,3-Dihydroxy-Isovalerate, Umbelliferone, and Naringenin, respectively. Data are presented as the mean ±SD (n = 3). *P* values for each comparison were indicated.


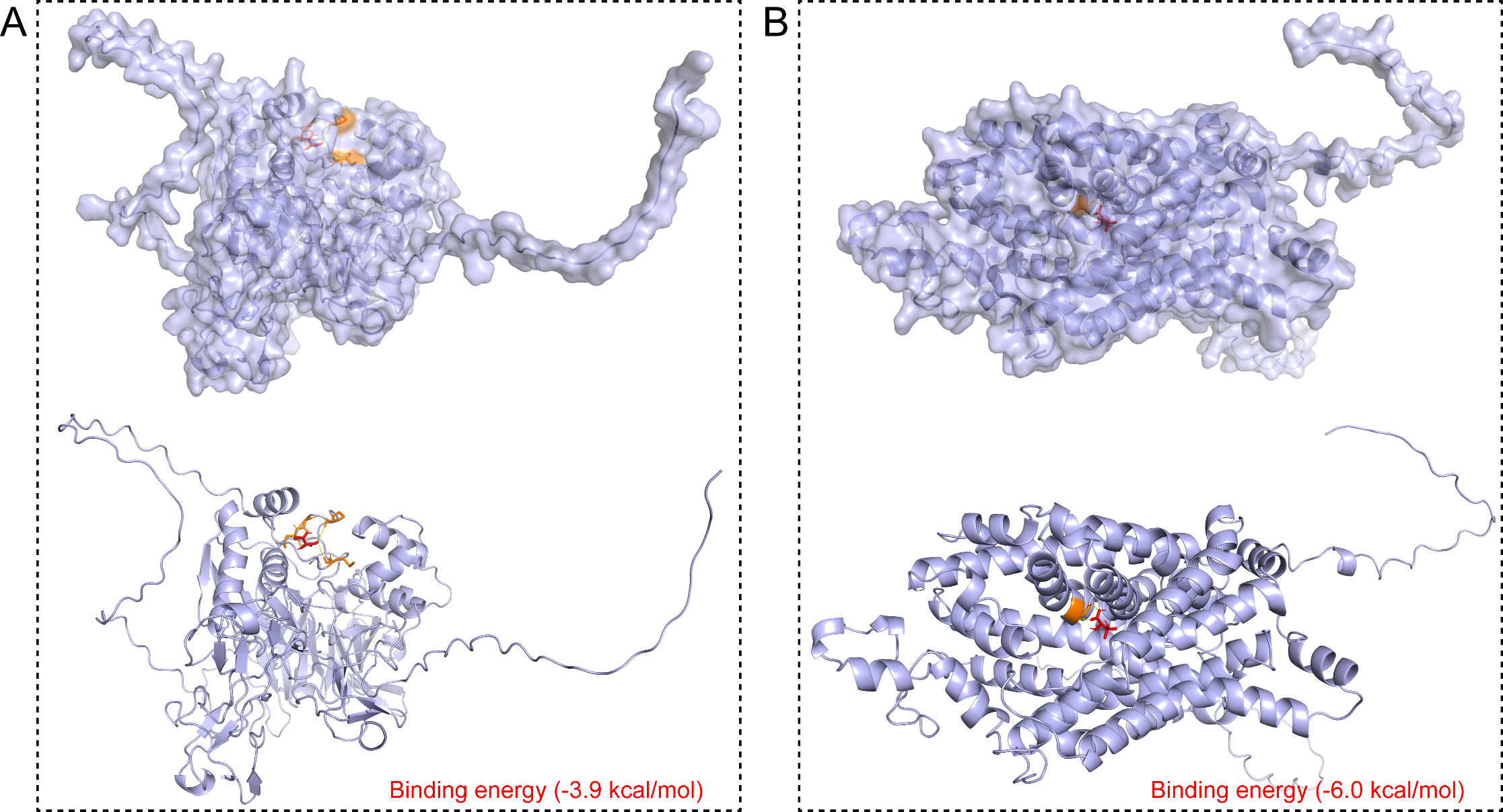


**Supplementary Figure 4.** Molecular docking of (R)-2,3-dihydroxy-isovalerate with MMP9 (A) and GAT-3 (B).


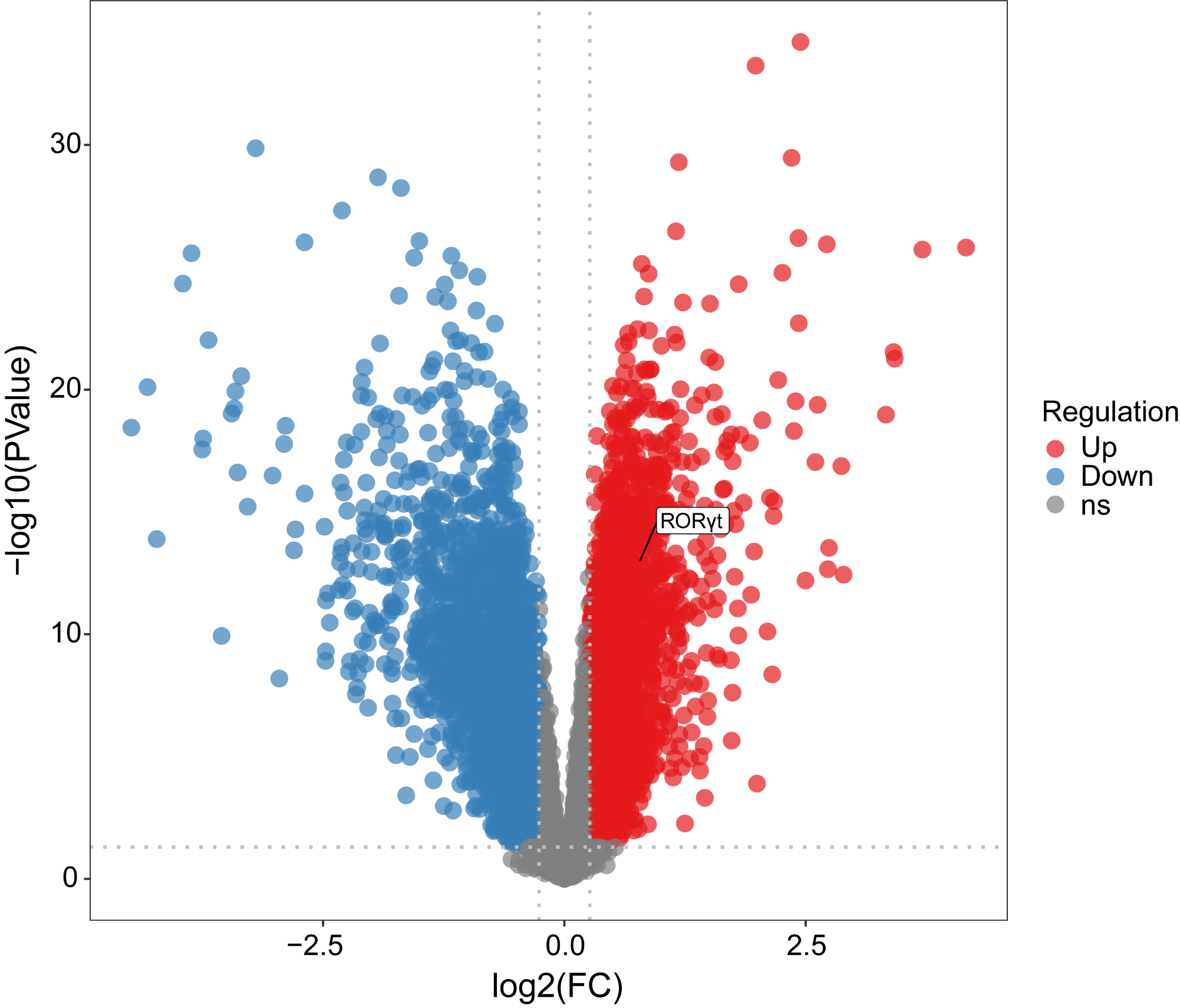


**Supplementary Figure 5.** Volcano plot showing differential gene expression from the GEO database (GSE59071).

**
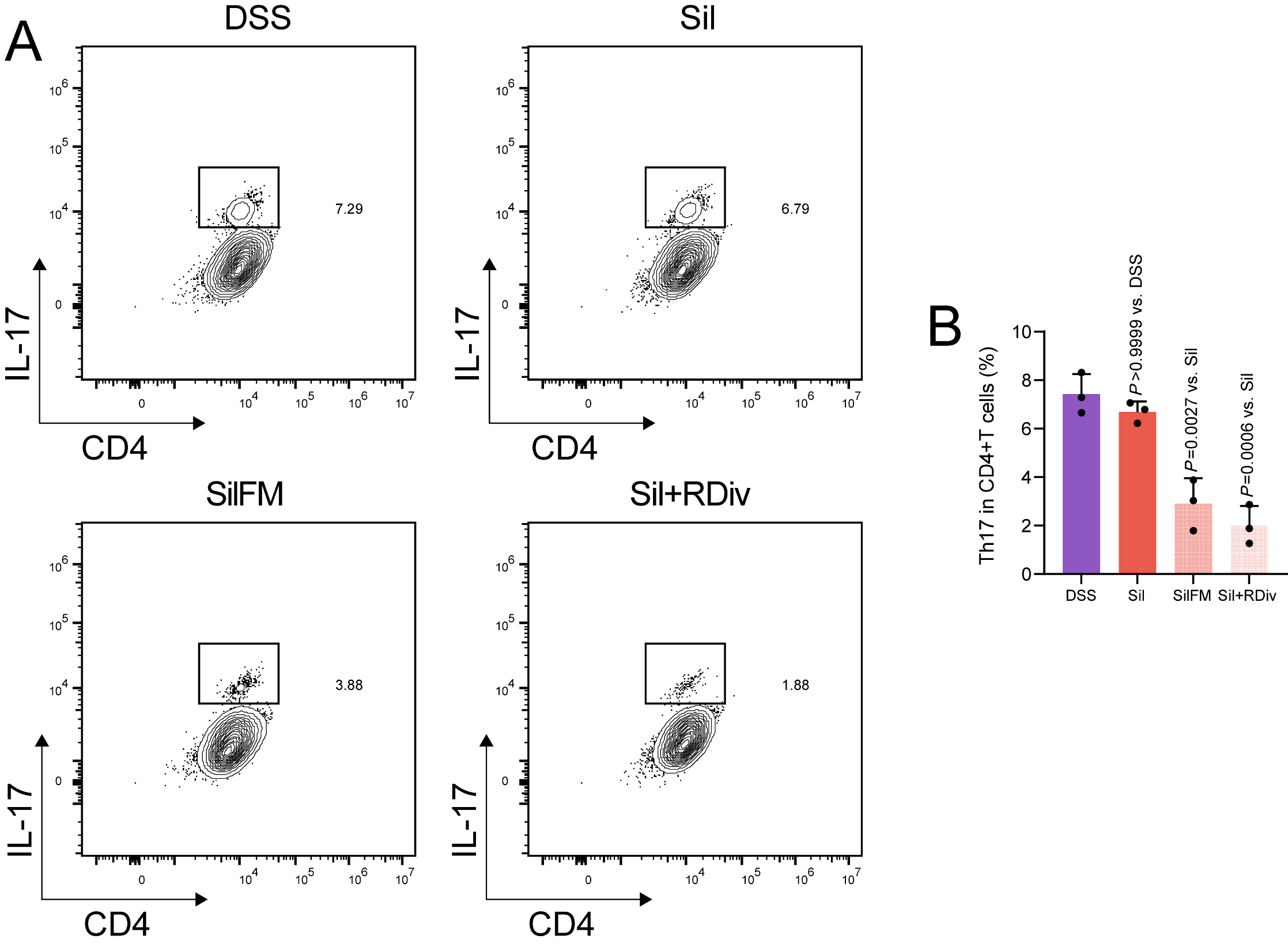
**

**Supplementary Figure 6.** (A, B) Characterization and quantitative analysis of colonic Th17 cell infiltration. Data are presented as the mean ± SD (n = 3). *P* values for each comparison were indicated.


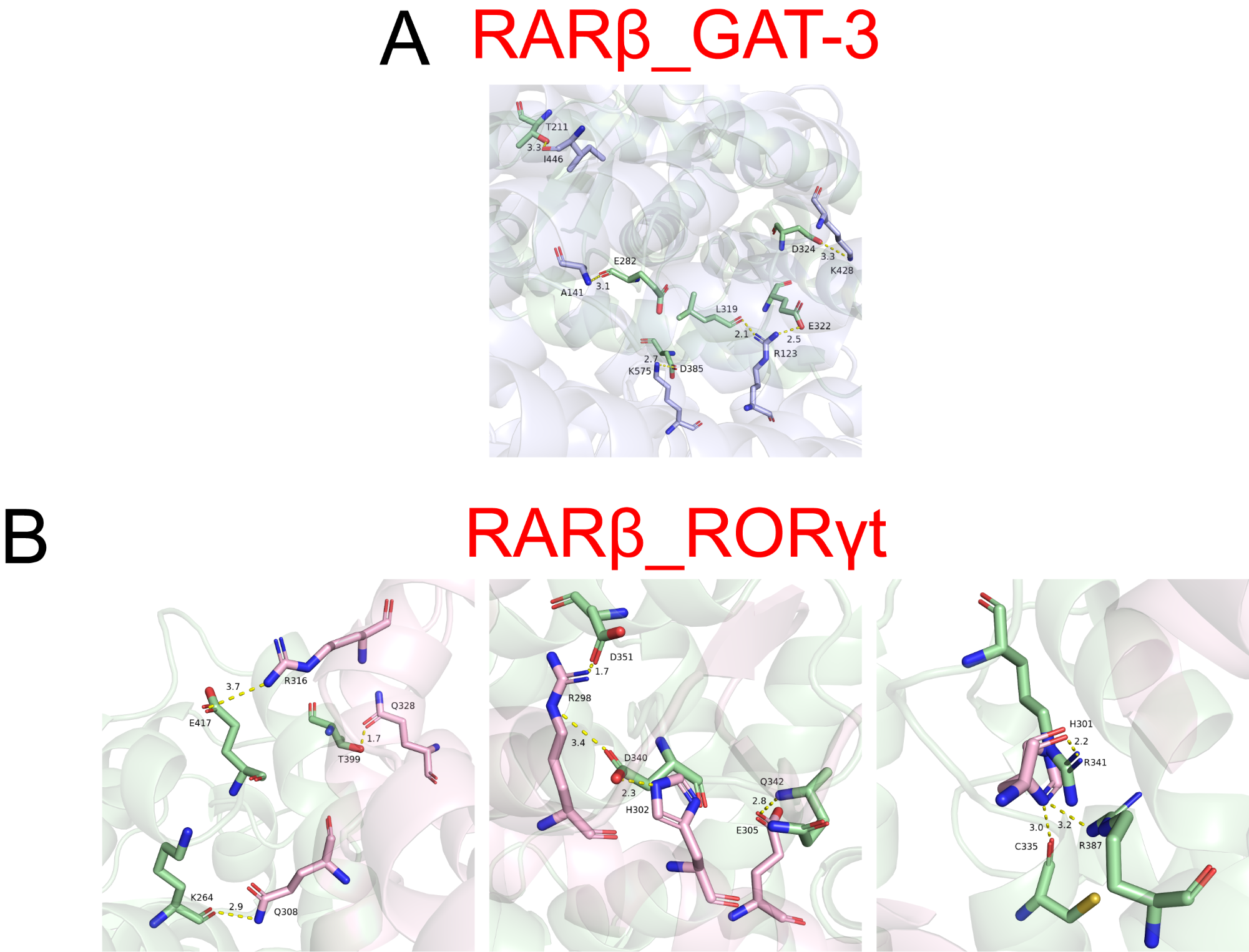


**Supplementary Figure 7.** Protein-protein docking. (A) Docking interaction between the GAT-3 protein and the RARβ protein. (B) Docking interaction between the RARβ protein and the RORγt protein.


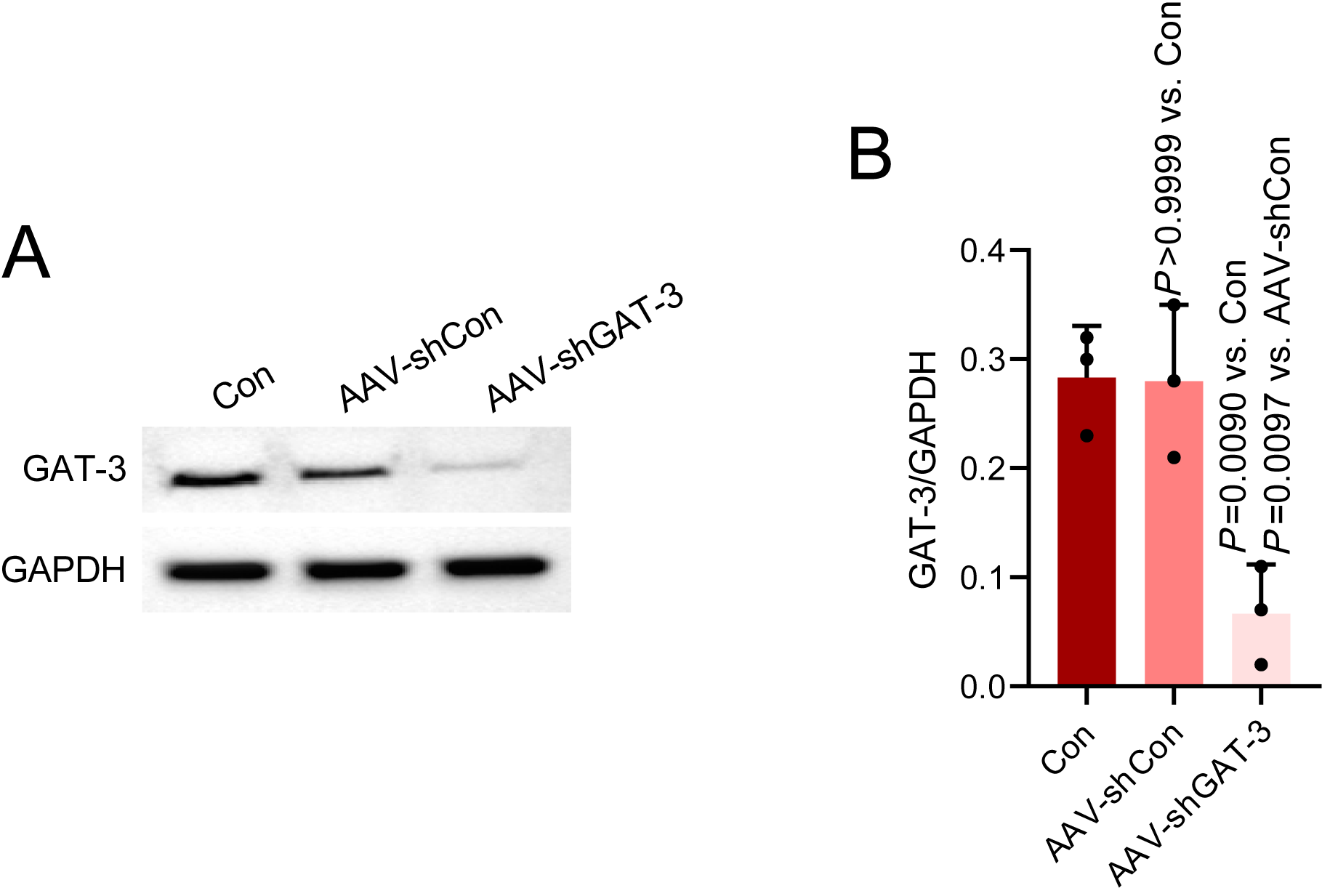


**Supplementary Figure 8.** Validation of the impact of AAV-shGAT-3 on the mouse colon. (A, B) GAT-3 expression and its semi-quantitative analysis in the colon. Data are presented as the mean ± SD (n = 3). *P* values for each comparison were indicated.

**Supplementary Table 1.** Scoring criteria of DAI

| **Weight loss (%)** | **Stool consistency** | **Rectal bleeding** | **DAI score** |
| --- | --- | --- | --- |
| None | Normal | Negative | 0 |
| 1–5 | Mild soft stool | Occult blood weakly positive (+) | 1 |
| 5–10 | Sever soft stool | Occult blood positive (++) | 2 |
| 10–20 | Mild diarrhea | Occult blood intensely positive (+++) | 3 |
| >20 | Severe diarrhea | Gross blood | 4 |

Note: The total score of these parameters was divided by three to calculate the DAI.
